# Genetic Recombination Is Targeted towards Gene Promoter Regions in Dogs

**DOI:** 10.1371/journal.pgen.1003984

**Published:** 2013-12-12

**Authors:** Adam Auton, Ying Rui Li, Jeffrey Kidd, Kyle Oliveira, Julie Nadel, J. Kim Holloway, Jessica J. Hayward, Paula E. Cohen, John M. Greally, Jun Wang, Carlos D. Bustamante, Adam R. Boyko

**Affiliations:** 1 Department of Genetics, Albert Einstein College of Medicine, Bronx, New York, New York, United States of America; 2 BGI-Shenzhen, Shenzhen, China; 3 Department of Human Genetics, University of Michigan, Ann Arbor, Michigan, United States of America; 4 Department of Biomedical Sciences, Cornell University College of Veterinary Medicine, Ithaca, New York, United States of America; 5 Department of Genetics, Stanford University, Stanford, California, United States of America; University of Cambridge, United Kingdom

## Abstract

The identification of the H3K4 trimethylase, PRDM9, as the gene responsible for recombination hotspot localization has provided considerable insight into the mechanisms by which recombination is initiated in mammals. However, uniquely amongst mammals, canids appear to lack a functional version of *PRDM9* and may therefore provide a model for understanding recombination that occurs in the absence of *PRDM9*, and thus how *PRDM9* functions to shape the recombination landscape. We have constructed a fine-scale genetic map from patterns of linkage disequilibrium assessed using high-throughput sequence data from 51 free-ranging dogs, *Canis lupus familiaris*. While broad-scale properties of recombination appear similar to other mammalian species, our fine-scale estimates indicate that canine highly elevated recombination rates are observed in the vicinity of CpG rich regions including gene promoter regions, but show little association with H3K4 trimethylation marks identified in spermatocytes. By comparison to genomic data from the Andean fox, *Lycalopex culpaeus*, we show that biased gene conversion is a plausible mechanism by which the high CpG content of the dog genome could have occurred.

## Author Summary

Recombination in mammalian genomes tends to occur within highly localized regions known as recombination hotspots. These hotspots appear to be a ubiquitous feature of mammalian genomes, but tend to not be shared between closely related species despite high levels of DNA sequence similarity. This disparity has been largely explained by the discovery of *PRDM9* as the gene responsible for localizing recombination hotspots via recognition and binding to specific DNA motifs. Variation within *PRDM9* can lead to changes to the recognized motif, and hence changes to the location of recombination hotspots thought the genome. Multiple studies have shown that *PRDM9* is under strong selective pressure, apparently leading to a rapid turnover of hotspot locations between species. However, uniquely amongst mammals, *PRDM9* appears to be dysfunctional in dogs and other canids. In this paper, we investigate how the loss of *PRDM9* has affected the fine-scale recombination landscape in dogs and contrast this with patterns seen in other species.

## Introduction

Until recently the mechanisms controlling the localization of recombination hotspots in mammalian genomes were largely unknown. However, recent research has revealed that zinc-finger protein, PRDM9, binds to specific DNA motifs in the early stages of recombination initiation in order to direct such events [1]–[3]. PRDM9 trimethylates lysine 4 of histone H3 (H3K4me3), an epigenetic modification specifically enriched around recombination initiation sites [4]–[6]. The importance of PRDM9 for the localization of recombination events has been demonstrated in both humans [7], [8] and mice [2], [6], with recent results in mice suggesting that PRDM9 determines the location of virtually all recombination hotspots in these organisms [6].

Variation in the zinc-finger encoding domain of *PRDM9* in humans can alter the DNA motif to which the protein binds, and in turn alter the activity of recombination hotspots [7]–[9]. High levels of variation in *PRDM9* across species [2], [10], [11] may explain why humans and chimpanzees do not share recombination hotspots despite very high levels of overall sequence identity [10]. Indeed, *PRDM9* shows clear evidence of rapid evolution across the metazoan taxa covering an era of roughly 700 million years [11]. Despite successful formation of double-strand breaks (DSBs) [6], the initiating event in meiotic recombination, knockout of *Prdm9* results in infertility in both male and female mice with arrest of spermatogenesis and oogenesis at pachynema, impairment of DSB repair, chromosome asynapsis, and disrupted sex-body formation in males [12]. Intriguingly, *Prdm9* has been shown to be involved with hybrid sterility, potentially implicating it in the process of speciation [12].

Given the wide-ranging importance of *PRDM9*, it was therefore surprising to note that dogs (*Canis familiaris*) and other canids are the only known mammals to carry functionally inert versions of *PRDM9* with multiple disruptive mutations [11], [13]. This implies that either the function of *PRDM9* is carried out by another gene, or that dogs have been able to avoid the loss of fertility associated with loss of *PRDM9* while also ensuring that recombination continues to occur.

In order to gain insight into recombination across the canine genome, we have constructed a genetic map using patterns of linkage disequilibrium (LD) estimated from next-generation sequencing of 51 dogs. Methods for estimating recombination rates from polymorphism data have been validated at both broad and fine scales [14]–[16], and have previously been used to obtain relatively broad-scale recombination rate estimates in dogs via the use of SNP microarray data [13]. A potential concern when using such methods in dogs is that breed-formation bottlenecks can lead to considerable levels of inbreeding. For this reason, we have utilized genetic polymorphism data collected primarily from free-ranging dogs from geographically diverse regions (Table S1) and largely lacking a history of excessive selective breeding following the original domestication event [17]. These non-breed dogs, which we term ‘village dogs’, show dramatically reduced levels of homozygosity, and a faster decay of LD when compared to inbred breed dogs (Figure S1).

## Results

The dogs were sequenced to 8–12× coverage with 101 bp paired end reads (Table S2), allowing identification of 13.6 million autosomal variants, and 366,000 variants on chromosome X. Based on comparisons to Illumina CanineHD microarray [18] SNP data, we estimate that we have >98% power to detect variants with a minor allele frequency of 5%, and a genotype accuracy of 99.1%. As estimation of genetic maps can be moderately sensitive to false-positive variant calls [10], we performed extensive variant filtering to identify a subset of high-quality variants (see Methods). Our filtered set consisted of 3.5 million autosomal SNPs, and 198,000 SNPs on the X chromosome, which we used to construct the genetic map.

**Figure 1:**
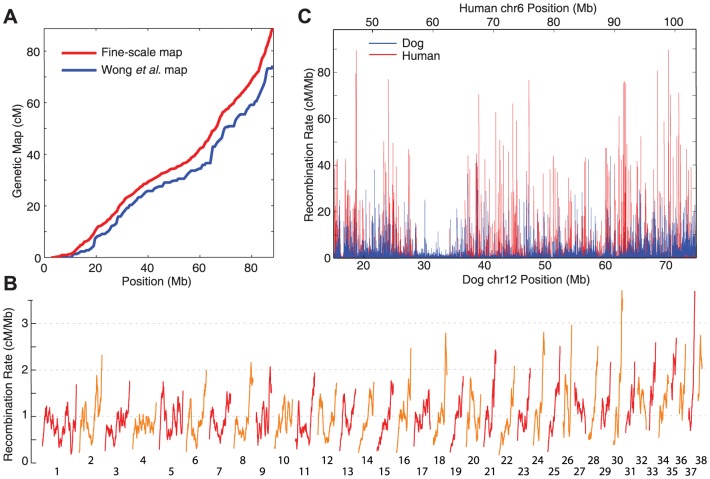
The distribution of recombination. A) Genetic maps for chromosome 2 estimated using LD (red) and pedigrees [19] (blue). Other chromosomes are shown in Figure S2. B) Genome-wide broad-scale recombination rates for each autosomal chromosome (red and orange). Rates were smoothed at the 5 Mb scale. C) Fine-scale recombination rates compared between dog (blue) and human (red) over a largely syntenic 60 Mb region [35].

To validate our recombination rate estimates, we compared our estimates to broad-scale experimental estimates obtained from pedigree studies [19]. There is strong agreement between our map and the linkage map at the broad scale (Pearson r = 0.87 at 5 Mb; Figure 1A, Figure S2, and Figure S3). Consistent with observations in other species [20], recombination rates tend to be highest in telomeric regions, and lowest near the centromere (Figure 1B). The correlation between chromosome physical length and total map length is similar for our map and the linkage map (Pearson r = 0.88 and r = 0.83 respectively; Figure S4). We conclude that our recombination rate estimates obtained from patterns of LD in population sequencing data are capable of accurately recapitulating the canine genetic map.

Our estimates suggest a more uniform distribution of recombination in the dog genome than has been seen in human (Figure S5A), as has been reported previously [13]. However, simulations indicate that the estimate of this distribution from patterns of linkage disequilibrium is sensitive to the effective population size, with larger effective population sizes leading to higher estimates of the background recombination rate (Figure S5B). As such, given that the effective population size estimated in dogs is larger than human, we cannot conclude the recombination is more uniformly distributed in dog.

Previous LD-based estimates of recombination in dog by Axelsson *et al.* were obtained using a microarray with 170k markers [13], with estimates averaged over multiple breeds. At the broad scale, there is good agreement between the two studies, which correlate more strongly with each other than the pedigree-based map (Figure S6). However, the increased marker density of our study allows investigation of the fine-scale recombination landscape in dogs. Despite the apparent loss of PRDM9 in dogs, we detect 7,677 hotspot-like peaks in the recombination rate throughout the canine genome, with a median width of 4.3 kb (Figure 1C).

We compared these hotspots to those identified by Axelsson *et al.* [13] and found we could confirm the presence of 1,090 out of the 4,074 hotspots identified by that study (27%). The overlap between the two sets is strongly significant (p<0.0001, assessed by 10,000 randomizations of the hotspot locations), suggesting both datasets are picking up on real signal. However, the relatively low concordance suggests either low power or an elevated false positive rate in one or both studies. To investigate further, we have plotted the average recombination rate as measured by the Axelsson study around hotspots identified by our study (Figure S7A). We see that recombination rate estimates tend to be higher for hotspots identified by both studies. However, hotspots identified by our study alone still show a peak in recombination in the Axelsson study, suggesting agreement between the two studies even when there was in sufficient power to call a hotspot in the Axelsson study. Conversely, the average recombination rate as measured by our study around hotspots identified by the Axelsson study (Figure S7B) shows only very weak elevation of recombination around hotspots not identified by our study.

### High recombination in CpG-rich regions

In humans and mice, specific DNA motifs have been implicated as the binding sites for *PRDM9* [1], [4], [21]. In order to investigate if DNA motifs could be identified within canine recombination hotspots, we selected 6,228 hotspot regions with no missing sequence data. For each hotspot we identified a region on the same chromosome showing no evidence for local recombination rate elevation (‘coldspots’), and with GC content within 0.5% of that of the hotspot, and CpG content within 0.1%. If more than one such region could be found, we selected the one that matched the hotspot most closely in terms of SNP density. In this way, we were able to identify 4,759 hotspots with matched coldspots.

Using the sequences of the matched hotspots and coldspots, we performed a search of motifs showing enrichment in hotspot sequences. Our results indicate an extremely strong association with CpG-rich motifs (Table 1), with the most significant motif being the 7-mer CGCCGCG (p = 1.1e-21, Fisher's Exact Test after Bonferroni correction), which is found in 21.3% of hotspots but only 13.2% of coldspots, a relative enrichment of 61%. These highly CpG-rich motifs retain significantly high levels of enrichment in hotspots having masked either repeat or non-repeat DNA sequence.

**Table 1:** Motifs enriched in hotspots.

Motif Length	Motif	Present in Hotspots	Present in Coldspots	Relative Enrichment	p-value (All Sequence)	p-value (Non-repeat Sequence)	p-value (Repeat Sequence)
6	CGCGCG	20.7%	13.5%	1.54	1.24E-17	4.32E-14	2.35E-04
6	CGCCGC	43.4%	35.8%	1.21	1.48E-10	3.31E-15	2.84E-05
6	CCGCGC	38.3%	31.2%	1.23	9.58E-10	6.69E-11	3.15E-09
6	CGCGGC	39.5%	33.5%	1.18	2.31E-06	1.67E-07	1.37E-04
6	CGGCCG	30.8%	25.8%	1.19	1.42E-04	1.25E-04	1.59E-03
6	CCGCCG	42.3%	36.9%	1.15	1.48E-04	1.10E-07	1.09E-03
7	CGCCGCG	21.3%	13.2%	1.61	1.14E-21	1.51E-15	7.83E-08
7	CCGCGCG	16.4%	9.8%	1.67	1.94E-17	1.10E-10	3.42E-05
7	CGCGCGC	14.8%	8.6%	1.72	4.88E-17	8.27E-11	7.98E-03
7	GCGCCGC	27.4%	19.3%	1.42	7.59E-17	6.31E-14	8.86E-04
7	CGCCCGC	25.8%	18.7%	1.38	4.68E-13	1.12E-09	2.36E-06
7	CCGCCGC	29.1%	21.9%	1.33	4.57E-12	6.69E-12	3.82E-05
7	CCGCGCC	24.9%	18.4%	1.36	6.13E-11	2.71E-05	9.50E-15
7	CGCGCCG	17.3%	11.7%	1.48	7.11E-11	3.10E-03	1.49E-09
7	CGCCGCC	31.7%	24.8%	1.28	5.37E-10	2.20E-11	1.57E-03
7	CCCGCGC	25.7%	19.4%	1.33	9.90E-10	1.58E-04	2.35E-11
7	CCGCGGC	25.4%	19.3%	1.32	6.24E-09	5.13E-06	8.39E-07
7	CCCGCGG	26.4%	20.8%	1.27	1.07E-06	3.65E-04	3.84E-05
7	GGCCGCC	33.0%	27.5%	1.20	6.01E-05	8.96E-04	7.69E-03
7	CGCGGGC	24.9%	20.0%	1.25	9.52E-05	4.00E-03	2.38E-03
7	CGGCGGC	26.2%	21.5%	1.22	5.02E-04	9.50E-03	2.11E-03
8	CGCCGCGC	11.1%	5.9%	1.89	1.26E-15	7.96E-09	2.36E-03
8	CCGCCGCG	12.9%	7.2%	1.78	1.72E-15	8.54E-07	9.78E-06
8	CCGCGGCG	11.3%	6.2%	1.82	4.78E-14	4.39E-07	2.00E-04
8	GCGCCCGC	12.2%	7.6%	1.61	1.08E-09	2.64E-04	8.49E-03
8	CGCGCCCC	14.1%	9.3%	1.52	1.22E-08	8.16E-04	5.13E-04
8	GCCGCCGC	16.9%	12.2%	1.39	1.53E-06	5.18E-03	7.36E-03
8	CCCGCCCC	34.2%	28.2%	1.21	7.94E-06	3.10E-05	1.07E-04
8	CCCCGCCC	36.1%	30.6%	1.18	3.08E-04	1.55E-05	1.42E-03
9	CGCCCCGCC	10.5%	5.6%	1.89	6.11E-14	2.82E-07	3.42E-03
9	CCCCGCCCC	26.5%	20.1%	1.32	2.10E-08	4.60E-06	2.27E-03

CpG-rich motifs are strongly enriched in canine recombination hotspots. Motifs were detected by comparing DNA sequences within 4,759 hotspots to the same number of coldspots, having matched for CpG content and SNP density. P-values are adjusted for multiple testing using the Bonferroni correction.

Both GC and CpG content show a strong association with canine recombination at fine scales (Figure S8A and B). However, CpG content shows a stronger correlation with recombination rate than GC content over multiple scales (r = 0.37 vs r = 0.25 respectively at 1 Mb; Figure S8C and D). If both measures are included as predictors in a multiple regression model, CpG content has a positive association, whereas GC content is negative (Table S3).

**Figure 2:**
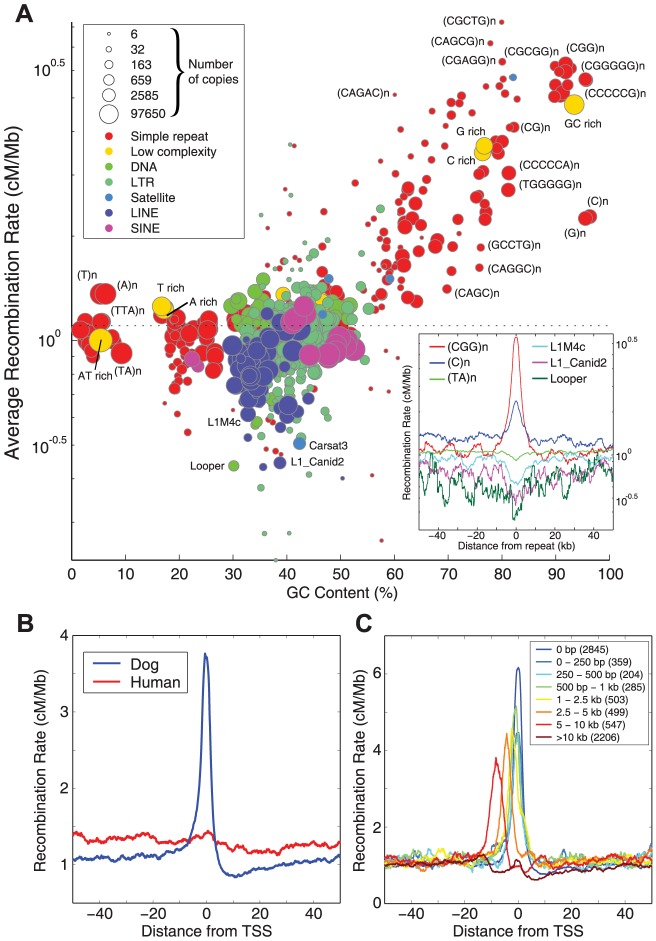
Recombination around genome features. A) Average recombination rates (on a logarithmic scale) for DNA repeats as a function of average GC content. Bubble size gives an indication of the number of repeats in a given family, with colors indicating higher-level repeat classes. Recombination rates for each repeat were estimated in a 5 kb window centered on the repeat, thinned so that no two repeats were within 10 kb. The insert shows (log scale) recombination rates around a selection of repeats. B) Recombination rates around TSS in dog (blue) shows an elevation that dwarfs the small elevation seen in human (red). C) Recombination rates around TSS partitioned on the basis of distance to the nearest CpG island (as defined by the UCSC genome browser), ranging from genes with a CpG island overlapping the TSS (dark blue) to genes with no CpG island within 10 kb (dark red).

The influence of GC and CpG content can also be seen when considering the average recombination rate around DNA repeats. The most recombinogenic repeats are low-complexity with high levels of GC and CpG content (Figure 2A). In contrast, the majority of LINE and SINE elements exhibit recombination rates close to the genome average, with a few such as Looper and L1_Canid2 showing weak suppression of recombination.

The association between recombination and CpG-dense regions is suggestive of an association with gene promoter regions. Indeed, we observe highly elevated rates of recombination around transcription start sites (TSS; Figure 2B and Figure S9), dwarfing the elevation that has been observed around TSS in humans and chimps [10], [22]. Of the 7,677 called hotspots, 29% overlap with a TSS, and 50% are within 14.7 kb. Only a small fraction (14%) of hotspots appear to be over 100 kb from a TSS. However, the elevation in recombination rate around TSS appears to be associated with CpG islands serving as promoter regions rather than the TSS themselves, as the recombination peak is shifted away from the TSS for genes with the nearest CpG island at some distance from the TSS (Figure 2C), with genes without a nearby CpG island not showing large peaks in local recombination. Conversely, CpG islands containing TSS show elevated recombination rates relative to CpG islands at some distance from TSS (Figure S10), although interestingly CpG islands >10 kb from the nearest TSS show higher rates than those near (but not containing) a TSS.

### Recombination has increased the CpG content of the dog genome

*PRDM9* is thought to have been disrupted early in canid evolution, as previous work has shown that the amino acid coding sequence contains multiple disruptive mutations across a diverse set of canid species [11], [13]. We have further investigated the extent of *PRDM9* disrupting mutations within the Canidae family by sequencing within exon containing the zinc-finger domain of *PRDM9* in the *Lycalopex* and *Urocyon* genera. Within in the Andean fox, *Lycalopex culpaeus* (6–7.4 Mya divergence from dogs [23]), we found the same disrupting frameshift mutation as has observed in dog (Table S4 and Table S5), as well as an additional frameshift, and a premature stop codon. In the Island fox, *Urocyon littoralis*, (>10 Mya divergence from dogs [23]), while we do not observe the same mutations seen in dog, we do observe a distinct premature stop codon, indicating that PRDM9 has been disrupted in this species as well. As none of the identified mutations are common to all species, it would appear that the original disruptive mutation likely occurred outside of sequenced exon.

Due to the early loss of *PRDM9*, it has been suggested that fine-scale patterns of recombination may be shared across species in the canid lineage [13], in contrast to other mammalian species in which hotspots are not shared [10]. Such inferences have been based on the effect of Biased Gene Conversion (BGC), in which a recombination-associated heteroduplex in the vicinity of an existing polymorphism can produce base-pair mismatches that are preferentially repaired with C/G alleles rather than A/T alleles (Figure S13). As such, BGC increases the probability of a C/G allele being transmitted to the next generation, and sustained BGC can ultimately alter the base composition of the genome [24].

To investigate if BGC is active around canine recombination hotspots, we consider the ratio of AT→GC polymorphisms to GC→AT polymorphisms within local regions of the genome (see Supplementary Text S1). In order to polarize polymorphisms in dog, we sequenced a female Andean Fox, which, as described above, is diverged from dogs by approximately 6–7.4 million years [23] and also lacks a functional version of *PRMD9*. The fox sample was sequenced to approximately 11× coverage using 100 bp paired-end Illumina sequencing. In the absence of a reference genome for this species, reads were mapped to the dog genome (canFam3.0). Using this data, we polarized the ancestral and derived alleles for polymorphisms observed in dog by assuming that shared alleles represent the ancestral allele. Using this data, we were able to polarize 3.2 million polymorphisms on the dog lineage with high confidence. Likewise, we performed SNP discovery in the fox data, and were able to identify and polarize 1.2 million polymorphisms on the fox linage.

**Figure 3:**
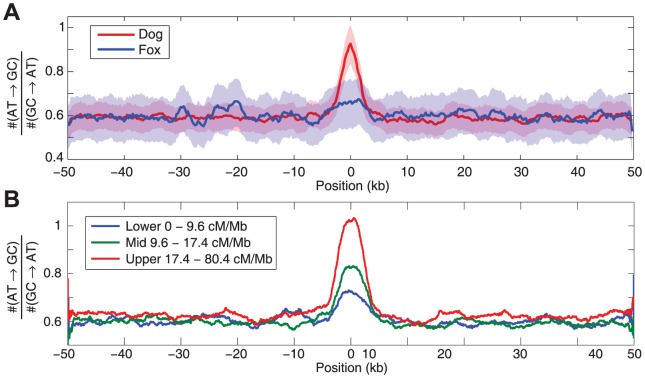
Evidence of biased gene conversion in dog and fox genomes. The plots show the ratio of the number of AT→GC polymorphisms relative to the number of GT→AT polymorphisms around hotspots detected in dog that were localized to within 5 kb. A) The (AT→GC)/(GC→AT) ratio around hotspots for SNPs discovered in dog (red) and fox (blue). 95% confidence intervals are shown as shaded areas, as assessed via bootstrap. B) The (AT→GC)/(GC→AT) ratio for polymorphisms originating along the dog lineage is stronger around more recombinogenic hotspots. The skew is shown around strong (red), intermediate (green), and weak (blue) hotspots, as defined by the peak rates described in the legend.

Around canine recombination hotspots, we observe a localized skew in the rate of AT to GC acquisition in the dog genome (Figure 3A), which is stronger for hotspots with higher peak recombination rates (Figure 3B; Figure S11A). The effect remains visible even after excluding all polymorphisms within putative CpG sites (Figure S11B). The dog genome is notable for its high density of CpG-rich regions [25], [26]. Given CpG-rich regions are highly recombinogenic in the dog genome, it is plausible that BGC is the mechanism by which the dog genome has acquired its high density of CpG rich regions. Specifically, if CpG regions are promoting recombination, and are thereby acquiring increasing levels of GC content via BGC, this would in turn further increase the CpG content of the region and hence further increase recombination in a self-reinforcing process.

If dogs and foxes shared the same hotspots, a similar pattern would be expected for polymorphisms observed on the fox linage. However, we do not see evidence of a skew in fox linage polymorphisms around canine recombination hotspots (Figure 3A), which would imply that these two species do not share recombination hotspots.

### The association between H3K4me3 and canine recombination

The role of PRDM9 is to trimethylate histone H3K4, and studies in mice have shown that nearly 95% of hotspots overlap an H3K4me3 mark [4]. It is therefore interesting to ask if canine recombination maintains an association with H3K4me3, especially given the apparent elevation of recombination around gene promoter regions in dogs. We have used ChIPseq to identify regions of H3K4me3 in dog spermatocytes during the leptotene/zygotene (L/Z) and pachytene phases of prophase I of meiosis. We identified 28,349 autosomal ChIPseq peaks in L/Z and 32,830 for pachynema. Of these, 8,721 (31%) peaks were unique to L/Z and 13,613 (41%) unique to pachynema.

**Figure 4:**
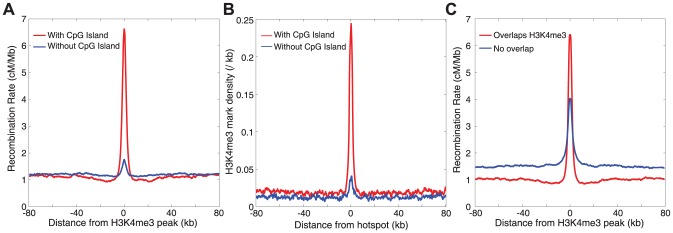
The relationship between recombination and H3K4 trimethylation. A) Recombination rates around H3K4me3 peaks, partitioned into peaks that contain a CpG island (red), and peaks without a CpG island (blue). B) The density of H3K4me3 marks around recombination hotspots partitioned into those hotspots that overlap a CpG island (red), and those that do not (blue). C) Recombination rates around CpG islands overlapping a H3K4me3 peak (red), and those not overlapping a H3K4me3 peak (blue).

While recombination rates do appear highly elevated around H3K4me3 marks, the effect is almost entirely explained by the presence or absence of a putative CpG island overlapping the mark (Figure 4A and B). The pattern is very similar for both L/Z and pachytene cells, albeit with a small increase in rate for pachytene-specific marks in the absence of CpG islands (Figure S12). Conversely, while CpG islands overlapping H3K4me3 marks are ∼60% more recombinogenic than those without marks, a strong elevation in local recombination rate remains visible for islands without H3K4me3 marks. Notably, putative CpG islands without H3K4me3 marks also appear to have elevated background rates (Figure 4C), and this effect persists even after extensive thinning CpG islands to ensure lack of clustering. This could reflect background sequence context, as islands with H3K4me3 marks have lower levels of CpG content in the flanking 50 kb than those without marks (2.2% vs 1.2%, p≪1e-16), or may be indicative of other epigenetic factors such as DNA methylation.

## Discussion

The apparent loss of *PRDM9* in canids makes dogs of particular interest for the study of meiotic recombination. Our study reveals that while broad scale patterns of recombination appear superficially typical of mammals, dogs appear to have a quite different landscape compared to other mammals so far studied at the fine scale. Of particular note is the strong association of recombination with CpG-rich features of the genome, particularly around promoter regions, which is reminiscent of the double-strand break localization around H3K4me3 marks that has been observed around promoter regions in *Prdm9*-knockout mice [6]. However, in contrast to these results, we find that the elevation in canine recombination rate around promoters appears to be primarily associated with CpG content, and shows little association with H3K4me3 marks. The association with promoter regions is also superficially reminiscent of the elevated recombination rates observed around promoter in yeast, which has been related to nucleosome spacing [27], [28].

The biased conversion of A/T to G/C alleles at already CpG enriched recombination hotspots may help explain the 2–3-fold increase in putative CpG islands in the dog genome compared to human and mouse [25]. Despite the relative high density of CpG islands in the dog genome, it has also been noted that dogs have fewer promoter-associated CpG islands than humans or mice, especially near essential and highly expressed genes [25]. If the role of *PRDM9* is to deflect DSBs away from functional elements, as has been suggested in other species [6], then the preferential loss of recombinogenic CpG islands near promoters in dogs may indicate that selection is acting to deflect recombination from genic regions since the loss of *PRDM9*.

*PRDM9*-knockout mice are infertile due to the failure to properly repair DSBs [29]. How canids escaped this infertility is unknown, but it must have occurred in the common ancestor of dogs, wolves, foxes and jackals, but after the split from Panda ∼49 Mya [13]. The stable recombination landscape of canids resulting from the loss of *PRMD9* may contribute to the ability to successfully hybridize relatively divergent canine species (e.g. dog and jackal [30]). Our comparison of substitution patterns in dogs and Andean foxes does not support the hypothesis that the death of *PRDM9* has resulted in the evolutionary stability of recombination along the canid lineage, at least at the fine scale. It is therefore possible that while *PRDM9* is dysfunctional, an unknown ortholog of *PRDM9* could have assumed a similar role.

Nonetheless, the observed recombination landscape in dogs does appear to have some unusual features, and it is plausible that these result directly from the loss of *PRDM9*. However, it is worth noting that in addition to the loss of *PRDM9*, canines are considerably diverged from other species that have so far been studied for fine-scale recombination rate variation, and it is plausible that the observed differences in the recombination landscape have also been influenced by other factors such as genomic structure. In order to fully understand the dynamics of recombination rate evolution, it will be necessary to obtain high-quality and fine-scale genetic maps across a wide range of species. As such maps become available, it will be possible to place the canine map into a proper evolutionary context, and thereby identify the factors that determine the forces that shape the distribution of recombination in the genome.

## Materials and Methods

DNA sequencing of 51 village dogs was performed using Illumina technology to 8–12 fold coverage, using 101 base-pair paired end reads (Table S1 and Table S2). Reads were aligned to the reference genome using *bwa* [31]. Variant calls were made using *GATK* [32], and phased using *BEAGLE* [33]. Extensive filters were applied to ensure that only high quality variants were used for the purposes of recombination rate estimation (see Supplementary Text S1 for details). After filtering, recombination rates were estimated using the statistical package, *LDhat* [15].

For H3K4me3 ChIPseq experiments, spermatocytes of various stages were cell types were purified sedimentation velocity (STA-PUT) of collagenase digested single cell suspensions. Chromatin immunoprecipitation (ChIP) of H3K4me3 was performed using standard procedures, and validated through qPCR (Table S6). Libraries were prepared using Tru-Seq adaptors, with sequencing performed using 150 bp paired-end reads from an Illumina HiSeq 2500 in Rapid Run mode. Reads were mapped to the canine reference genome, and H3K4me3 peaks called using *MACS* [34].

Detailed methods are available in the Supplementary Information. Genetic maps and called hotspots are available for download from: http://autonlab.einstein.yu.edu/dog_recomb/

### Ethics statement

Canine blood samples were collected under Cornell Institutional Animal Care and Use Committee (IACUC) approval (#2005-0151, 2007-0076 and 2011-0061) in accordance with applicable federal, state, and local laws, regulations, policies, and guidelines. For each animal, approximately 3–5 ml of blood was drawn from the cephalic vein into K2-EDTA blood collection tubes. Dogs were unsedated to minimize handling time and overall distress during the short blood drawing procedure. Culpeo blood was extracted from a captive female fox using a similar procedure during a routine physical by trained veterinary personnel at the Universidad de San Antonio Abad Zoo in Cusco.

## Supporting Information

Figure S1 Decay of LD for village dogs compared to breed dogs. Genotype LD was calculated between the village dogs (red hued lines), breed dogs (blue hued lines), and wolves (green line). The genotype data for wolves and breed dogs was taken from Axelsson *et al.* [13]. For the purposes of comparison, the sample size in each group was N = 6, and only sites present on the Illumina SNP microarray used by the Axelsson study were used. LD was calculated for sites with MAF>10%.
(EPS)

Figure S2 Genetic maps for all autosomes estimated using LD (red) and pedigrees [19] (blue).
(EPS)

Figure S3 Correlation at the 5 Mb scale between LD-based recombination rate estimates and those obtained from with Wong *et al* [*19*]. Each chromosome is indicated with a different marker.
(EPS)

Figure S4 A) Correlation between genetic map length and chromosome length for the LD-based map (black) and the linkage (red). Lines indicate least-squares lines of best fit. B) Shorter chromosomes tend to have higher recombination rates in both maps. Colors as for (A). Lines indicate a line of best fit estimated for a cubic function.
(EPS)

Figure S5 A) The distribution of recombination for each dog chromosome, compared to the human estimates from HapMap. B) Conclusions regarding differences between the human and dog distributions should be treated with caution due to sensitivity in the estimator to the effective population size. This figure shows the distribution of recombination for data simulated with three different effective population sizes (Ne).
(EPS)

Figure S6 Pearson correlation at various scales between recombination rate estimates from this study, Axelsson *et al.* [13], and Wong *et al.* [19].
(EPS)

Figure S7 A) Recombination rate estimates from the Axelsson *et al.* study around hotspots identified by this study. Estimates are shown for all hotspots (blue), those called by both studies (green), and those not called by both studies (red). B) Rate estimates from this study around hotspots identified in the Axelsson *et al.* study. Colors as for (A).
(EPS)

Figure S8 The association between recombination and GC and CpG content. A) GC content verse normalized recombination rate at 1 kb, 10 kb, and 100 kb scales. Recombination rates are shown for dog (red) and human (blue), having normalized the rates in each species by subtracting off the mean and dividing by the standard deviation. B) As for (A), but showing CpG content verses normalized recombination rate. C) Pearson correlation of GC content with (un-normalized) recombination rate, as a function of scale. D) As for (C) but showing correlation between CpG content and recombination rate at various scales.
(EPS)

Figure S9 Recombination in each region of a gene. Shown are recombination estimates 50 kb Upstream of the transcription start site (TSS), within the 1^st^ Exon, 1^st^ Intron, Middle Exon, Last Intron, Last Exon, and 50 kb Downstream from the transcription end site (TES). Estimates within each intron and exon were estimated across 50 equally spaced bins, allowing a percentage to express the distance through the intron/exon.
(EPS)

Figure S10 Recombination rates around CpG islands partitioned on the basis of distance to the nearest transcription start site.
(EPS)

Figure S11 A) No evidence of biased gene conversion around recombination coldspots. The plots show the ratio of the number of AT→GC mutations relative to the number of GT→AT mutations for polymorphisms identified in dog (red) and fox (blue). B) The same ratio around hotspots and coldspots in dogs, having excluded all polymorphisms for which either allele would create CpG dinucleotide.
(EPS)

Figure S12 Recombination rates for H3K4me3 peaks overlapping CpG islands (A) and not overlapping CpG islands (B). The three lines represent peaks shared between pachytene and L/Z (blue), those unique to pachytene (green), and those unique to L/Z (red).
(EPS)

Figure S13 A) Cartoon representation of a possible biased gene conversion mechanism. A double-strand break in the vicinity of a G/A polymorphism results in the partial loss of the A/T base pair on the 2^nd^ chromosome. The subsequent repair following strand invasion results in mis-pairing between the G and T nucleotides. Biased gene conversion results from the biased repair of this mismatch, which favors the G allele over the T allele. B) Behavior of the ‘skew’ statistic in 5000 simulated hotspots, shown for simulations including biased gene conversion (red), and without biased gene conversion (blue).
(EPS)

Table S1 Locations of collected samples.
(PDF)

Table S2 Details of sequence coverage.
(PDF)

Table S3 Predictors of recombination rate. Regression of recombination with GC content, CpG content, and gene density at various scales.
(PDF)

Table S4 *PRDM9* exon 7 DNA sequences. This table shows the ∼651 bp of DNA sequence from exon 7 of *PRDM9* in 24 species or breeds. Sequences in black were taken from Axelsson *et al.* [13] whereas the sequences from this study are highlighted in red. Sequences were aligned in MEGA version 5.05, specifically using the clustalW algorithm with standard parameters. Frameshift mutations are visible at positions 40 and 533 for the Andean Fox (Culpeo) respectively, with a stop codon starting at position 130. The island fox has a stop codon starting at position 607.
(PDF)

Table S5 PRDM9 exon 7 Amino Acid sequences. Data is as for Table S4, but translated to amino acid sequence. The frameshift mutations in the Andean Fox (Culpeo) can be seen at positions 14 and 178, with the stop codon at position 44. The Island Fox stop codon can be seen at position 203.
(PDF)

Table S6 Primers used for qPCR validation of ChIP.
(PDF)

Text S1 Details of supplementary methods and analyses.
(PDF)
