# Supplementary material for: Genetic Recombination Is Targeted towards Gene Promoter Regions in Dogs
Source: PLoS Genet. 2013 Dec 12;9(12):e1003984. doi: 10.1371/journal.pgen.1003984 (PMC3861134; doi:10.1371/journal.pgen.1003984)
Supplement: Table S1 — Locations of collected samples. (PDF) [file pgen.1003984.s014.pdf]

Table S1: Locations of collected samples

Population/region	N	Individuals
China (north)	8	Xilin Basset, Chow chow, Shar pei, Sichuan Liangshan dog, Sichuan Qingchuan dog, Chongqing dog, Guizhou Xiasi dog, Chinese field dog
China (south)	6	Kunming dog, Shandong canine, Shaanxi canine, Hebei canine, Mongolia canine, Kazakhstan shepherd dog
Europe	6	Labrador retriever, Caucasian shepherd, Portugal village dog (2), Croatia village dog, Bosnia village dog
India	6	Tibetan mastiff/shepherd mix, village dog (5)
Mideast	8	Afghan hound, Egypt village dog (2), Lebanon village dog (3), Qatar village dog (2)
Oceania	7	Borneo village dog (3), Papua New Guinea village dog (3), Taiwan village dog
Vietnam	6	Village dog (6)
Other	4	Xoloitzcuintl, Namibia village dog (3)
