# Supplementary material for: Genetic Recombination Is Targeted towards Gene Promoter Regions in Dogs
Source: PLoS Genet. 2013 Dec 12;9(12):e1003984. doi: 10.1371/journal.pgen.1003984 (PMC3861134; doi:10.1371/journal.pgen.1003984)
Supplement: Table S2 — Details of sequence coverage. (PDF) [file pgen.1003984.s015.pdf]

**Table S2: Details of sequence coverage**

| Dog ID | Population | Mean Autosomal Coverage |
|---|---|---|
| 1735 | Afghan Hound | 6.89 |
| 2972 | Labrador Retriever | 9.05 |
| 4669 | Xoloitzcuintli | 13.97 |
| BA19 | Bosnia (Tornjak) | 7.10 |
| Dog01 | China | 7.41 |
| Dog02 | China | 8.13 |
| Dog03 | China | 7.97 |
| Dog04 | China | 7.41 |
| Dog06 | China | 7.89 |
| Dog07 | China | 6.62 |
| Dog08 | China | 7.80 |
| Dog09 | China | 7.41 |
| Dog10 | China | 7.85 |
| Dog11 | China | 7.46 |
| Dog12 | China | 7.89 |
| Dog13 | China | 8.22 |
| Dog14 | China | 7.91 |
| Dog15 | China | 7.70 |
| EG44 | Egypt | 7.79 |
| EG49 | Egypt | 7.01 |
| HR85 | Bosnia (Istrian Shorthaired Hound) | 7.73 |
| HR93 | Bosnia (Caucasian Ovcharka) | 9.13 |
| ID125 | India | 12.66 |
| ID137 | India | 7.20 |
| ID165 | India | 9.47 |
| ID168 | India (Tibetan Masiff mix) | 8.34 |
| ID60 | India | 13.02 |
| ID91 | India | 7.50 |
| IN18 | Borneo | 4.94 |
| IN23 | Borneo | 6.95 |
| IN29 | Borneo | 5.74 |
| LB74 | Lebanon | 8.37 |
| LB79 | Lebanon | 8.76 |
| LB85 | Lebanon | 8.61 |
| NA63 | Namibia | 7.62 |
| NA8 | Namibia | 12.77 |
| NA89 | Namibia | 7.08 |
| PG115 | Papua New Guinea | 8.03 |
| PG122 | Papua New Guinea | 5.75 |
| PG84 | Papua New Guinea | 15.00 |
| PT61 | Portugal | 15.68 |
| PT71 | Portugal | 13.77 |
| QA27 | Qatar | 12.38 |
| QA5 | Qatar | 6.06 |
| TW04 | Taiwan | 14.94 |
| VN21 | Vietnam | 9.04 |
| VN37 | Vietnam | 6.77 |
| VN4 | Vietnam | 9.10 |
| VN42 | Vietnam | 9.08 |
| VN59 | Vietnam | 8.64 |
| VN76 | Vietnam | 6.11 |
