# Supplementary material for: Genetic Recombination Is Targeted towards Gene Promoter Regions in Dogs
Source: PLoS Genet. 2013 Dec 12;9(12):e1003984. doi: 10.1371/journal.pgen.1003984 (PMC3861134; doi:10.1371/journal.pgen.1003984)
Supplement: Table S3 — Predictors of recombination rate. Regression of recombination with GC content, CpG content, and gene density at various scales. (PDF) [file pgen.1003984.s016.pdf]

Table S3: Predictors of recombination rate

Correlation (Pearson coefficient, r)

	GC content	CpG content	Genes*
1kb	0.2571	0.3428	-0.0284
10kb	0.3082	0.4104	-0.0688
100kb	0.3162	0.3967	-0.0616
1Mb	0.2534	0.368	-0.0679

Multiple Regression with GC and CpG content

**Coefficient (b)**	constant	GC	CpG
1kb	5.43E-04	-6.71E-07	5.41E-04
10kb	0.0139	-0.0003	0.0089
100kb	0.1376	-0.0026	0.0731
1Mb	2.6939	-0.0627	0.9279
**p-value**	constant	GC	CpG
1kb	0	0.0071	0
10kb	0	1.83E-264	0
100kb	3.23E-62	9.69E-27	0.00E+00
1Mb	2.10E-27	1.12E-17	1.03E-54

Multiple Regression with GC, CpG, and gene content

**Coefficient (b)**	constant	GC	CpG	Genes*
1kb	5.53E-04	-1.55E-07	5.41E-04	-1.19E-04
10kb	0.0142	-0.0003	0.009	-0.0039
100kb	0.1367	-0.0023	0.0741	-0.0388
1Mb	2.2791	-0.047	0.8654	-0.6019
**p-value**	constant	GC	CpG	Genes*
1kb	0	0.5347	0	0
10kb	0	4.33E-236	0	0
100kb	1.71E-62	1.86E-22	0.00E+00	1.00E-84
1Mb	2.39E-19	6.71E-10	9.30E-48	1.24E-10

Multiple Regression with GC, GC^2, CpG, and gene content

**Coefficient (b)**	constant	GC	GC^2	CpG	Genes*
1kb	-0.0015	9.96E-05	-1.25E-06	6.48E-04	-1.15E-04
10kb	-0.0522	0.0029	-3.89E-05	0.0113	-0.0038
100kb	-0.6816	0.0369	-4.77E-04	0.1017	-0.0423
1Mb	-6.2314	0.3676	-0.0052	1.1839	-0.7575
**p-value**	constant	GC	GC^2	CpG	Genes*
1kb	0	0	0	0	0
10kb	0	0	0	0	0
100kb	5.07E-124	5.67E-165	1.70E-191	0	9.07E-104
1Mb	2.62E-12	5.02E-18	4.67E-23	9.71E-69	3.91E-16

* Fraction of bin between transcription start end sites
