# Supplementary material for: Genetic Recombination Is Targeted towards Gene Promoter Regions in Dogs
Source: PLoS Genet. 2013 Dec 12;9(12):e1003984. doi: 10.1371/journal.pgen.1003984 (PMC3861134; doi:10.1371/journal.pgen.1003984)
Supplement: Table S4 — PRDM9 exon 7 DNA sequences. This table shows the ∼651 bp of DNA sequence from exon 7 of PRDM9 in 24 species or breeds. Sequences in black were taken from Axelsson et al. [13] whereas the sequences from this study are highlighted in red. Sequences were aligned in MEGA version 5.05, specifically using the clustalW algorithm with standard parameters. Frameshift mutations are visible at positions 40 and 533 for the Andean Fox (Culpeo) respectively, with a stop codon starting at position 130. The island fox has a stop codon starting at position 607. (PDF) [file pgen.1003984.s017.pdf]

Table S4: PRDM9 exon 7 DNA sequences

Sequence	1–10	11–20	21–30	31–40	41–50	51–60	61–70	71–78
JF750638.1|Canis_lupus_familiaris_Beagle	CCTAACCCAG	AGATACATCC	ATGTCCATCC	TGCTCTCTG-	CCTTCTCCAG	TCAGAAATTC	CTCAGCCAGC	ATTTGGAA
JF750639.1|Canis_lupus_familiaris_Portugese_Water_Dog	CCTAACCCAG	AGATACATCC	ATGTCCATCC	TGCTCTCTG-	CCTTCTCCAG	TCAGAAATTC	CTCAGCCAGC	ATTTGGAA
JF750640.1|Canis_lupus_familiaris_Bearded_Collie	CCTAACCCAG	AGATACATCC	ATGTCCATCC	TGCTCTCTG-	CCTTCTCCAG	TCAGAAATTC	CTCAGCCAGC	ATTTGGAA
JF750641.1|Canis_lupus_familiaris_Standard_Poodle	CCTAACCCAG	AGATACATCC	ATGTCCATCC	TGCTCTCTG-	CCTTCTCCAG	TCAGAAATTC	CTCAGCCAGC	ATTTGGAA
JF750642.1|Canis_lupus_familiaris_Boxer	CCTAACCCAG	AGATACATCC	ATGTCCATCC	TGCTCTCTG-	CCTTCTCCAG	TCAGAAATTC	CTCAGCCAGC	ATTTGGAA
JF750643.1|Canis_lupus_familiaris_Dalmatian	CCTAACCCAG	AGATACATCC	ATGTCCATCC	TGCTCTCTG-	CCTTCTCCAG	TCAGAAATTC	CTCAGCCAGC	ATTTGGAA
JF750644.1|Canis_lupus_familiaris_Labrador_retriever	CCTAACCCAG	AGATACATCC	ATGTCCATCC	TGCTCTCTG-	CCTTCTCCAG	TCAGAAATTC	CTCAGCCAGC	ATTTGGAA
JF750645.1|Canis_lupus_familiaris_German_Shepard	CCTAACCCAG	AGATACATCC	ATGTCCATCC	TGCTCTCTG-	CCTTCTCCAG	TCAGAAATTC	CTCAGCCAGC	ATTTGGAA
JF750646.1|Canis_lupus_familiaris_Rough_Collie	CCTAACCCAG	AGATACATCC	ATGTCCATCC	TGCTCTCTG-	CCTTCTCCAG	TCAGAAATTC	CTCAGCCAGC	ATTTGGAA
JF750647.1|Canis_lupus_familiaris_Eurasier	CCTAACCCAG	AGATACATCC	ATGTCCATCC	TGCTCTCTG-	CCTTCTCCAG	TCAGAAATTC	CTCAGCCAGC	ATTTGGAA
JF750648.1|Canis_lupus_familiaris_Polish_Lowland_Sheepdog	CCTAACCCAG	AGATAC????	????CCATCC	TGCTCTCTG-	CCTTCTCCAG	TCAGAAATTC	CTCAGCCAGC	ATTTGGAA
JF750649.1|Canis_lupus_familiaris_Rottweiler	CCTAACCCAG	AGATACATCC	ATGTCCATCC	TGCTCTCTG-	CCTTCTCCAG	TCAGAAATTC	CTCAGCCAGC	ATTTGGAA
JF750650.1|Canis_lupus_familiaris_Smalandsstrovare	CCTAACCCAG	AGATACATCC	ATGTCCATCC	TGCTCTCTG-	CCTTCTCCAG	TCAGAAATTC	CTCAGCC?GC	ATTTGGAA
JF750651.1|Canis_lupus_familiaris_Swedish_Elkhound	CCTAACCCAG	AGATAC????	????CCATCC	TGCTCTCTG-	CCTTCTCCAG	TCAGAAATTC	CTCAGCCAGC	ATTTGGAA
JF750652.1|Canis_lupus_familiaris_Nova_Scotia_Duck_Tolling_Retriever	CCTAACCCAG	AGATACATCC	ATGTCCATCC	TGCTCTCTG-	CCTTCTCCAG	TCAGAAATTC	CTCAGCCAGC	ATTTGGAA
JF750653.1|Canis_lupus_isolate_ref21	CCTAACCCAG	AGATACATCC	ATGTCCATCC	TGCTCTCTG-	CCTTCTCCAG	TCAGAAATTC	CTCAGCCAGC	ATTTGGAA
JF750654.1|Canis_lupus_isolate_ref34	CCTAACCCAG	AGATACATCC	ATGTCCATCC	TGCTCTCTG-	CCTTCTCCAG	TCAGAAATTC	CTCAGCCAGC	ATTTGGAA
JF750655.1|Vulpes_vulpes	CCTAACCCAG	AGATATATCC	ATGTCCATCC	TGCTCTCTGT	CCTTCTCCAG	TCAGAAATTC	CTCAGCCAGC	ATTTGGAA
JF750656.1|Speothos_venaticus	CCTAACCCAG	AGATACATCC	ATGTCCATCC	TGCTCTCTG-	CCTTCTCCAG	TCAGAAATTC	CTCAGCCAGC	ATTTGGAA
JF750657.1|Lycaon_pictus	CCTAACCCAG	AGATACATCC	ATGTCCATCC	TGCTCTCTG-	CCTTCTCCAG	TCAGAAATTC	CTCAGCCAGC	ATTTGGAA
JF750658.1|Canis_mesomelas	CCTAACCCAG	AGATACATCC	ATGTCCATCC	TGCTCTCTG-	CCTTCTCCAG	TCAGAAATTC	CTCAGCCAGC	ATTTGGAA
JF750659.1|Canis_aureus	CCTAACCCAG	AGATACATCC	ATGTCCATCC	TGCTCTCTG-	CCTTCTCCAG	TCAGAAATTC	CTCAGCCAGC	ATTTGGAA
Island_Fox|Urocyon littoralis	CYTAACCCAG	AGATACATCC	ATGTCCATCC	TGCTCTCTGG	CCTTCTCCAG	TCAGAAATTC	CTCAGCCAGC	ATTTGGAA
Culpeo|Lycalopex culpaeus	CCTAACCCAG	AGATACATCC	ATGTCCATCC	TGCTCTCTG-	CCTTCTCCAG	TCAGAAATTC	CTCAGCCAGC	ATTTGGAA

Sequence	79–88	89–98	99–108	109–118	119–128	129–138	139–148	149–156
JF750638.1|Canis_lupus_familiaris_Beagle	CACAAYCACC	CTTCTCAGAT	CCTCCCACGA	ATATCTGTAA	GAGAACATTT	CCGACCAAAA	GATCCATGCC	CAGGTTGT
JF750639.1|Canis_lupus_familiaris_Portugese_Water_Dog	CACAATCACC	CTTCTCAGAT	CCTCCCACGA	ATATCTGTAA	GAGAACATTT	CCGACCAAAA	GATCCATGCC	CAGGTTGT
JF750640.1|Canis_lupus_familiaris_Bearded_Collie	CACAATCACC	CTTCTCAGAT	CCTCCCACGA	ATATCTGTAA	GAGAACATTT	CCGACCAAAA	GATCCATGCC	CAGGTTGT
JF750641.1|Canis_lupus_familiaris_Standard_Poodle	CACAATCACC	CTTCTCAGAT	CCTCCCACGA	ATATCTGTAA	GAGAACATTT	CCGACCAAAA	GATCCATGCC	CAGGTTGT
JF750642.1|Canis_lupus_familiaris_Boxer	CACAATCACC	CTTCTCAGAT	CCTCCCACGA	ATATCTGTAA	GAGAACATTT	CCGACCAAAA	GATCCATGCC	CAGGTTGT
JF750643.1|Canis_lupus_familiaris_Dalmatian	CACAATCACC	CTTCTCAGAT	CCTCCCACGA	ATATCTGTAA	GAGAACATTT	CCGACCAAAA	GATCCATGCC	CAGGTTGT
JF750644.1|Canis_lupus_familiaris_Labrador_retriever	CACAATCACC	CTTCTCAGAT	CCTCCCACGA	ATATCTGTAA	GAGAACATTT	CCGACCAAAA	GATCCATGCC	CAGGTTGT
JF750645.1|Canis_lupus_familiaris_German_Shepard	CACAACCACC	CTTCTCAGAT	CCTCCCACGA	ATATCTGTAA	GAGAACATTT	CCGACCAAAA	GATCCATGCC	CAGGTTGT
JF750646.1|Canis_lupus_familiaris_Rough_Collie	CACAATCACC	CTTCTCAGAT	CCTCCCACGA	ATATCTGTAA	GAGAACATTT	CCGACCAAAA	GATCCATGCC	CAGGTTGT
JF750647.1|Canis_lupus_familiaris_Eurasier	CACAATCACC	CTTCTCAGAT	CCTCCCACGA	ATATCTGTAA	GAGAACATTT	CCGACCAAAA	GATCCATGCC	CAGGTTGT
JF750648.1|Canis_lupus_familiaris_Polish_Lowland_Sheepdog	CACAATCACC	CTTCTCAGAT	CCTCCCACGA	ATATCTGTAA	GAGAACATTT	CCGACCAAAA	GATCCATGCC	CAGGTTGT
JF750649.1|Canis_lupus_familiaris_Rottweiler	CACAAYCACC	CTTCTCAGAT	CCTCCCACGA	ATATCTGTAA	GAGAACATTT	CCGACCAAAA	GATCCATGCC	CAGGTTGT
JF750650.1|Canis_lupus_familiaris_Smalandsstrovare	CACAATCACC	CTTCTCAGAT	CCTCCCACGA	ATATCTGTAA	GAGAACATTT	CCGACCAAAA	GATCCATGCC	CAGGTTGT
JF750651.1|Canis_lupus_familiaris_Swedish_Elkhound	CACAAYCACC	CTTCTCAGAT	CCTCCCACGA	ATATCTGTAA	GAGAACATTT	CCGACCAAAA	GATCCATGCC	CAGGTTGT
JF750652.1|Canis_lupus_familiaris_Nova_Scotia_Duck_Tolling_Retriever	CACAATCACC	CTTCTCAGAT	CCTCCCACGA	ATATCTGTAA	GAGAACATTT	CCGACCAAAA	GATCCATGCC	CAGGTTGT
JF750653.1|Canis_lupus_isolate_ref21	CACAATCACC	CTTCTCAGAT	CCTCCCACGA	ATATCTGTAA	GAGAACATTT	CCGACCAAAA	GATCCATGCC	CAGGTTGT
JF750654.1|Canis_lupus_isolate_ref34	CACAATCACC	CTTCTCAGAT	CCTCCCACGA	ATATCTGTAA	GAGAACATTT	CCGACCAAAA	GATCCATGCC	CAGGTTGT
JF750655.1|Vulpes_vulpes	CACAATCACC	CTTCTCAGAT	CCTCCCACGA	ATATCTATAA	GAGAACATTT	CCAACCAAAA	GATCCATGCC	CAGGTTGT
JF750656.1|Speothos_venaticus	CACAATCACC	CTTCTCAGAT	CCTCCCATGA	ATATCTGTAA	GAGAACATTT	CCGACCAAAA	GATCCATGCC	CAGGTTGT
JF750657.1|Lycaon_pictus	CACAATCACC	CTTCTCAGAT	CCTCCCACGA	ATATCTGTAA	GAGAACATTT	CCGACCAAAA	GATCCATGCC	CAGGTTGT
JF750658.1|Canis_mesomelas	CACAATCACC	CTTCTCAGAT	CCTCCCACAA	ATATCTGTAA	GAGAACATTT	CCGACCAAAA	GATCCATGCC	CAGGTTGT
JF750659.1|Canis_aureus	CACAATCACC	CTTCTCAGAT	CCTCCCACGA	ATATCTGTAA	GAGAACATTT	CCGACCAAAA	GATCCATGCC	CAGGTTGT
Island_Fox|Urocyon littoralis	CACAATCACC	CTTMTCAGAT	CCTCCCACGA	ATATCTGTAA	GAGAACATTT	CCGACCAAAA	GATCCATGCC	CAGGTTGT
Culpeo|Lycalopex culpaeus	CACAATCACC	CTTCTCAGAT	CCTCCCACGA	ATATCTGTAA	GAGAACATTT	CTGACCAAAA	GATCCATGCC	CAGGTTGT

Sequence	157–166	167–176	177–186	187–196	197–206	207–216	217–226	227–234
JF750638.1|Canis_lupus_familiaris_Beagle	CAGAATCAGC	AGCAGCAGCA	ACATTCTGAT	CCACAGCGCT	GGAATGACAG	AGCTAAAGGT	CAAGAGGGCA	AAGAAAGG
JF750639.1|Canis_lupus_familiaris_Portugese_Water_Dog	CAGAATCAGC	AGCAGCAGCA	ACATTCTGAT	CCACAGCGCT	GGAATGACAG	AGCTAAAGGT	CAAGAGGGCA	AAGAAAGG
JF750640.1|Canis_lupus_familiaris_Bearded_Collie	CAGAATCAGC	AGCAGCAGCA	ACATTCTGAT	CCACAGCGCT	GGAATGACAG	AGCTAAAGGT	CAAGAGGGCA	AAGAAAGG
JF750641.1|Canis_lupus_familiaris_Standard_Poodle	CAGAATCAGC	AGCAGCAGCA	ACATTCTGAT	CCACAGCGCT	GGAATGACAG	AGCTAAAGGT	CAAGAGGGCA	AAGAAAGG
JF750642.1|Canis_lupus_familiaris_Boxer	CAGAATCAGC	AGCAGCAGCA	ACATTCTGAT	CCACAGCGCT	GGAATGACAG	AGCTAAAGGT	CAAGAGGGCA	AAGAAAGG
JF750643.1|Canis_lupus_familiaris_Dalmatian	CAGAATCAGC	AGCAGCAGCA	ACATTCTGAT	CCACAGCGCT	GGAATGACAG	AGCTAAAGGT	CAAGAGGGCA	AAGAAAGG
JF750644.1|Canis_lupus_familiaris_Labrador_retriever	CAGAATCAGC	AGCAGCAGCA	ACATTCTGAT	CCACAGCGCT	GGAATGACAG	AGCTAAAGGT	CAAGAGGGCA	AAGAAAGG
JF750645.1|Canis_lupus_familiaris_German_Shepard	CAGAATCAGC	AGCAGCAGCA	ACATTCTGAT	CCACAGCGCT	GGAATGACAG	AGCTAAAGGT	CAAGAGGGCA	AAGAAAGG
JF750646.1|Canis_lupus_familiaris_Rough_Collie	CAGAATCAGC	AGCAGCAGCA	ACATTCTGAT	CCACAGCGCT	GGAATGACAG	AGCTAAAGGT	CAAGAGGGCA	AAGAAAGG
JF750647.1|Canis_lupus_familiaris_Eurasier	CAGAATCAGC	AGCAGCAGCA	ACATTCTGAT	CCACAGCGCT	GGAATGACAG	AGCTAAAGGT	CAAGAGGGCA	AAGAAAGG
JF750648.1|Canis_lupus_familiaris_Polish_Lowland_Sheepdog	CAGAATCAGC	AGCAGCAGCA	ACATTCTGAT	CCACAGCGCT	GGAATGACAG	AGCTAAAGGT	CAAGAGGGCA	AAGAAAGG
JF750649.1|Canis_lupus_familiaris_Rottweiler	CAGAATCAGC	AGCAGCAGCA	ACATTCTGAT	CCACAGCGCT	GGAATGACAG	AGCTAAAGGT	CAAGAGGGCA	AAGAAAGG
JF750650.1|Canis_lupus_familiaris_Smalandsstrovare	CAGAATCAGC	AGCAGCAGCA	ACATTCTGAT	CCACAGCGCT	GGAATGACAG	AGCTAAAGGT	CAAGAGGGCA	AAGAAAGG
JF750651.1|Canis_lupus_familiaris_Swedish_Elkhound	CAGAATCAGC	AGCAGCAGCA	ACATTCTGAT	CCACAGCGCT	GGAATGACAG	AGCTAAAGGT	CAAGAGGGCA	AAGAAAGG
JF750652.1|Canis_lupus_familiaris_Nova_Scotia_Duck_Tolling_Retriever	CAGAATCAGC	AGCAGCAGCA	ACATTCTGAT	CCACAGCGCT	GGAATGACAG	AGCTAAAGGT	CAAGAGGGCA	AAGAAAGG
JF750653.1|Canis_lupus_isolate_ref21	CAGAATCAGC	AGCAGCAGCA	ACATTCTGAT	CCACAGCGCT	GGAATGACAG	AGCTAAAGGT	CAAGAGGGCA	AAGAAAGG
JF750654.1|Canis_lupus_isolate_ref34	CAGAATCAGC	AGCAGCAGCA	ACATTCTGAT	CCACAGCGCT	GGAATGACAG	AGCTAAAGGT	CAAGAGGGCA	AAGAAAGG
JF750655.1|Vulpes_vulpes	CAGAATCAGC	AGCAGCAGCA	ACATTCTGAT	CCACAGTGCT	GGAATGACAG	AGCTAAAGGT	CAAGAGGGCA	AAGAAAGG
JF750656.1|Speothos_venaticus	CAGAATCAGC	AGCAGCAGCA	ACATTCTGAT	CCACAGCGCT	GGAATGACAG	AGCTAAAGGT	CAAGAGGGCA	AAGAAAGG
JF750657.1|Lycaon_pictus	CAGAATCAGC	AGCAGCAGCA	ACATTCTGAT	CCACAGCGCT	GGAGTGACAG	AGCTAAAGGT	CAAGAGGGCA	AAGAAAGG
JF750658.1|Canis_mesomelas	CAGAATCAGC	AGCAGCAGCA	ACATTCTGAT	CCACAGCGCT	GGAATGACAG	AGCTAAAGGT	CAAGAGGGCA	AAGAAAGG
JF750659.1|Canis_aureus	CAGAATCAGC	AGCAGCAGCA	ACATTCTGAT	CCACAGCGCT	GGAATGACAG	AGCTAAAGGT	CAAGAGGGCA	AAGAAAGG
Island_Fox|Urocyon littoralis	CAGAATCAGC	AGCAGCAGCA	ACATTCTGAT	CCACAGCGCT	GGAATGACAG	AGCTAAAGGT	CAAGAGGGCA	AAGAAAGG
Culpeo|Lycalopex culpaeus	CAGAATCAGC	AGCAGCAGCA	ACATTCTGAT	CCACAGCGCT	GGAATGACAG	AGCTAAAGGT	CAAGAGGGCA	AAGAAAGG

Sequence	235–244	245–254	255–264	265–274	275–284	285–294	295–304	305–312
JF750638.1|Canis_lupus_familiaris_Beagle	TTCAAACCTT	TGCCTAAAAG	TATAAGGCAG	AGAAGAATTT	CAAGGGCCTT	TTCCACCCCG	TGCAAAGGGC	AAACAACG
JF750639.1|Canis_lupus_familiaris_Portugese_Water_Dog	TTCAAACCTT	TGCCTAAAAG	TATAAGGCAG	AGAAGAATTT	CAAGGGCCTT	TTCCACCCCG	TGCAAAGGGC	AAACAACG
JF750640.1|Canis_lupus_familiaris_Bearded_Collie	TTCAAACCTT	TGCCTAAAAG	TATAAGGCAG	AGAAGAATTT	CAAGGGCCTT	TTCCACCCCG	TGCAAAGGGC	AAACAACG
JF750641.1|Canis_lupus_familiaris_Standard_Poodle	TTCAAACCTT	TGCCTAAAAG	TATAAGGCAG	AGAAGAATTT	CAAGGGCCTT	TTCCACCCCG	TGCAAAGGGC	AAACAACG
JF750642.1|Canis_lupus_familiaris_Boxer	TTCAAACCTT	TGCCTAAAAG	TATAAGGCAG	AGAAGAATTT	CAAGGGCCTT	TTCCACCCCG	TGCAAAGGGC	AAACAACG
JF750643.1|Canis_lupus_familiaris_Dalmatian	TTCAAACCTT	TGCCTAAAAG	TATAAGGCAG	AGAAGAATTT	CAAGGGCCTT	TTCCACCCCG	TGCAAAGGGC	AAACAACG
JF750644.1|Canis_lupus_familiaris_Labrador_retriever	TTCAAACCTT	TGCCTAAAAG	TATAAGGCAG	AGAAGAATTT	CAAGGGCCTT	TTCCACCCCG	TGCAAAGGGC	AAACAACG
JF750645.1|Canis_lupus_familiaris_German_Shepard	TTCAAACCTT	TGCCTAAAAG	TATAAGGCAG	AGAAGAATTT	CAAGGGCCTT	TTCCACCCCG	TGCAAAGGGC	AAACAACG
JF750646.1|Canis_lupus_familiaris_Rough_Collie	TTCAAACCTT	TGCCTAAAAG	TATAAGGCAG	AGAAGAATTT	CAAGGGCCTT	TTCCACCCCG	TGCAAAGGGC	AAACAACG
JF750647.1|Canis_lupus_familiaris_Eurasier	TTCAAACCTT	TGCCTAAAAG	TATAAGGCAG	AGAAGAATTT	CAAGGGCCTT	TTCCACCCCG	TGCAAAGGGC	AAACAACG
JF750648.1|Canis_lupus_familiaris_Polish_Lowland_Sheepdog	TTCAAACCTT	TGCCTAAAAG	TATAAGGCAG	AGAAGAATTT	CAAGGGCCTT	TTCCACCCCG	TGCAAAGGGC	AAACAACG
JF750649.1|Canis_lupus_familiaris_Rottweiler	TTCAAACCTT	TGCCTAAAAG	TATAAGGCAG	AGAAGAATTT	CAAGGGCCTT	TTCCACCCCG	TGCAAAGGGC	AAACAACG
JF750650.1|Canis_lupus_familiaris_Smalandsstrovare	TTCAAACCTT	TGCCTAAAAG	TATAAGGCAG	AGAAGAATTT	CAAGGGCCTT	TTCCACCCCG	TGCAAAGGGC	AAACAACG
JF750651.1|Canis_lupus_familiaris_Swedish_Elkhound	TTCAAACCTT	TGCCTAAAAG	TATAAGGCAG	AGAAGAATTT	CAAGGGCCTT	TTCCACCCCG	TGCAAAGGGC	AAACAACG
JF750652.1|Canis_lupus_familiaris_Nova_Scotia_Duck_Tolling_Retriever	TTCAAACCTT	TGCCTAAAAG	TATAAGGCAG	AGAAGAATTT	CAAGGGCCTT	TTCCACCCCG	TGCAAAGGGC	AAACAACG
JF750653.1|Canis_lupus_isolate_ref21	TTCAAACCTT	TGCCTAAAAG	TATAAGGCAG	AGAAGAATTT	CAAGGGCCTT	TTCCACCCCG	TGCAAAGGGC	AAACAACG
JF750654.1|Canis_lupus_isolate_ref34	TTCAAACCTT	TGCCTAAAAG	TATAAGGCAG	AGAAGAATTT	CAAGGGCCTT	TTCCACCCCG	TGCAAAGGGC	AAACAACG
JF750655.1|Vulpes_vulpes	TTCAAACCTT	TGCCTAAA-G	TATAAGGCAG	AGAAAAATTT	CAAGGGCCTT	TTCCACCCCG	TGCAAAGGAC	AAACAACG
JF750656.1|Speothos_venaticus	TTCAAACCTT	TGCCTAAACG	TATAAGGCAG	AGAAGAATTT	CAAGGGCCTT	TTCCACCCCG	TGCAAAGGGC	AAACAACG
JF750657.1|Lycaon_pictus	TTCAAACCTT	TGCCTAAAAG	TATAAGGCAG	AGAAGAATTT	CAAGGGCCTT	TTCCACCCCG	TGCAAAGGGC	AAACAACG
JF750658.1|Canis_mesomelas	TTCAAACCTT	TGCCTAAAAG	TATAAGGCAG	AGAAGAATTT	CAAGGGCCTT	TTCCACCCCG	TGCAAAGGGC	AAACAACG
JF750659.1|Canis_aureus	TTCAAACCTT	TGCCTAAAAG	TATAAGGCAG	AGAAGAATTT	CAAGGGCCTT	TTCCACCCCG	TGCAAAGGGC	AAACAACG
Island_Fox|Urocyon littoralis	TTCAAACCTT	TGCCTAAAAG	TATAAGGCAG	AGAAGAATTT	CAAGGGCCTT	TTCTACCCCG	TGCAAAGGAC	AAACAACG
Culpeo|Lycalopex culpaeus	TTCAAACCTT	TGCCTAAACG	TATAAGGCAG	AGAAGAATTT	CAAGAGCCTT	TTCCACCCCG	TGCAAAGGGC	AAACAACG

Sequence	313–322	323–332	333–342	343–352	353–362	363–372	373–382	383–390
JF750638.1|Canis_lupus_familiaris_Beagle	TGTGAGGGAA	TAGTGAAGGA	AGAGCCCAGC	GCAGGCTCAC	AGAAATTGAA	TCCAGAGGAC	ACAGGCAAAT	TATTCAAG
JF750639.1|Canis_lupus_familiaris_Portugese_Water_Dog	TGTGAGGGAA	TAGTGAAGGA	AGAGCCCAGC	GCAGGCTCAC	AGAAATTGAA	TCCAGAGGAC	ACAGGCAAAT	TATTCAAG
JF750640.1|Canis_lupus_familiaris_Bearded_Collie	TGTGAGGGAA	TAGTGAAGGA	AGAGCCCAGC	GCAGGCTCAC	AGAAATTGAA	TCCAGAGGAC	ACAGGCAAAT	TATTCAAG
JF750641.1|Canis_lupus_familiaris_Standard_Poodle	TGTGAGGGAA	TAGTGAAGGA	AGAGCCCAGC	GCAGGCTCAC	AGAAATTGAA	TCCAGAGGAC	ACAGGCAAAT	TATTCAAG
JF750642.1|Canis_lupus_familiaris_Boxer	TGTGAGGGAA	TAGTGAAGGA	AGAGCCCAGC	GCAGGCTCAC	AGAAATTGAA	TCCAGAGGAC	ACAGGCAAAT	TATTCAAG
JF750643.1|Canis_lupus_familiaris_Dalmatian	TGTGAGGGAA	TAGTGAAGGA	AGAGCCCAGC	GCAGGCTCAC	AGAAATTGAA	TCCAGAGGAC	ACAGGCAAAT	TATTCAAG
JF750644.1|Canis_lupus_familiaris_Labrador_retriever	TGTGAGGGAA	TAGTGAAGGA	AGAGCCCAGC	GCAGGCTCAC	AGAAATTGAA	TCCAGAGGAC	ACAGGCAAAT	TATTCAAG
JF750645.1|Canis_lupus_familiaris_German_Shepard	TGTGAGGGAA	TAGTGAAGGA	AGAGCCCAGC	GCAGGCTCAC	AGAAATTGAA	TCCAGAGGAC	ACAGGCAAAT	TATTCAAG
JF750646.1|Canis_lupus_familiaris_Rough_Collie	TGTGAGGGAA	TAGTGAAGGA	AGAGCCCAGC	GCAGGCTCAC	AGAAATTGAA	TCCAGAGGAC	ACAGGCAAAT	TATTCAAG
JF750647.1|Canis_lupus_familiaris_Eurasier	TGTGAGGGAA	TAGTGAAGGA	AGAGCCCAGC	GCAGGCTCAC	AGAAATTGAA	TCCAGAGGAC	ACAGGCAAAT	TATTCAAG
JF750648.1|Canis_lupus_familiaris_Polish_Lowland_Sheepdog	TGTGAGGGAA	TAGTGAAGGA	AGAGCCCAGC	GCAGGCTCAC	AGAAATTGAA	TCCAGAGGAC	ACAGGCAAAT	TATTCAAG
JF750649.1|Canis_lupus_familiaris_Rottweiler	TGTGAGGGAA	TAGTGAAGGA	AGAGCCCAGC	GCAGGCTCAC	AGAAATTGAA	TCCAGAGGAC	ACAGGCAAAT	TATTCAAG
JF750650.1|Canis_lupus_familiaris_Smalandsstrovare	TGTGAGGGAA	TAGTGAAGGA	AGAGCCCAGC	GCAGGCTCAC	AGAAATTGAA	TCCAGAGGAC	ACAGGCAAAT	TATTCAAG
JF750651.1|Canis_lupus_familiaris_Swedish_Elkhound	TGTGAGGGAA	TAGTGAAGGA	AGAGCCCAGC	GCAGGCTCAC	AGAAATTGAA	TCCAGAGGAC	ACAGGCAAAT	TATTCAAG
JF750652.1|Canis_lupus_familiaris_Nova_Scotia_Duck_Tolling_Retriever	TGTGAGGGAA	TAGTGAAGGA	AGAGCCCAGC	GCAGGCTCAC	AGAAATTGAA	TCCAGAGGAC	ACAGGCAAAT	TATTCAAG
JF750653.1|Canis_lupus_isolate_ref21	TGTGAGGGAA	TAGTGAAGGA	AGAGCCCAGC	GCAGGCTCAC	AGAAATTGAA	TCCAGAGGAC	ACAGGCAAAT	TATTCAAG
JF750654.1|Canis_lupus_isolate_ref34	TGTGAGGGAA	TAGTGAAGGA	AGAGCCCAGC	GCAGGCTCAC	AGAAATTGAA	TCCAGAGGAC	ACAGGCAAAT	TATTCAAG
JF750655.1|Vulpes_vulpes	TGTGAGGGAA	TAGTGAAGGA	AGAGCCCAGC	GCAGGCTCAC	AGAAATTGAA	TCCAGAGGAC	ACAGGCAAAT	TATTCAAG
JF750656.1|Speothos_venaticus	TGTGAGGGAA	TAGTGAAGGA	AGAGCCCAGC	GCAAGCTCAC	AGAAATTGAA	TCCAGAGGAY	ACAGGCAAAT	TATTCAAG
JF750657.1|Lycaon_pictus	TGTGAGGGAA	TAGTGAAGGA	AGAGCCCAGC	GCAGGCTCAC	AGAAATTGAA	TCCAGAGGAC	ACAGGCAAAT	TATTCAAG
JF750658.1|Canis_mesomelas	TGTGAGGGAA	TAGTGAAGGA	AGAGCCCAGC	GCAGGCTCAC	AGAAATTGAA	TCCAGAGGAC	ACAGGCAAAT	TATTCAAG
JF750659.1|Canis_aureus	TGTGAGGGAA	TAGTGAAGGA	AGAGCCCAGC	GCAGGCTCAC	AGAAATTGAA	TCCAGAGGAC	ACAGGCAAAT	TATTCAAG
Island_Fox|Urocyon littoralis	TGTGAGGGAA	TAGTGAAGGA	AGAGCCCAGC	ACAGGCTCAC	AGAAATTGAA	TCCAGAGGAC	ACAGGCAAAT	TATTCAAG
Culpeo|Lycalopex culpaeus	TGTGAGGGAA	TAGTGAAGGA	AGAGCCCAGC	GCAGGCTCAC	AGAAATTGAA	TCCAGAGGAC	ACAGGCAAAT	TATTCAAG

Sequence	391–400	401–410	411–420	421–430	431–440	441–450	451–460	461–468
JF750638.1|Canis_lupus_familiaris_Beagle	GGGGTAGGAA	TGACAAGAAT	TATAAGAGTC	AAATATAGAG	GCTGTGGGCG	AGGCTTCAAT	GATAGGTCAC	ATCTCAGC
JF750639.1|Canis_lupus_familiaris_Portugese_Water_Dog	GGGGTAGGAA	TGACAAGAAT	TATAAGAGTC	AAATATAGAG	GCTGTGGGCG	AGGCTTCAAT	GATAGGTCAC	ATCTCAGC
JF750640.1|Canis_lupus_familiaris_Bearded_Collie	GGGGTAGGAA	TGACAAGAAT	TATAAGAGTC	AAATATAGAG	GCTGTGGGCG	AGGCTTCAAT	GATAGGTCAC	ATCTCAGC
JF750641.1|Canis_lupus_familiaris_Standard_Poodle	GGGGTAGGAA	TGACAAGAAT	TATAAGAGTC	AAATATAGAG	GCTGTGGGCG	AGGCTTCAAT	GATAGGTCAC	ATCTCAGC
JF750642.1|Canis_lupus_familiaris_Boxer	GGGGTAGGAA	TGACAAGAAT	TATAAGAGTC	AAATATAGAG	GCTGTGGGCG	AGGCTTCAAT	GATAGGTCAC	ATCTCAGC
JF750643.1|Canis_lupus_familiaris_Dalmatian	GGGGTAGGAA	TGACAAGAAT	TATAAGAGTC	AAATATAGAG	GCTGTGGGCG	AGGCTTCAAT	GATAGGTCAC	ATCTCAGC
JF750644.1|Canis_lupus_familiaris_Labrador_retriever	GGGGTAGGAA	TGACAAGAAT	TATAAGAGTC	AAATATAGAG	GCTGTGGGCG	AGGCTTCAAT	GATAGGTCAC	ATCTCAGC
JF750645.1|Canis_lupus_familiaris_German_Shepard	GGGGTAGGAA	TGACAAGAAT	TATAAGAGTC	AAATATAGAG	GCTGTGGGCG	AGGCTTCAAT	GATAGGTCAC	ATCTCAGC
JF750646.1|Canis_lupus_familiaris_Rough_Collie	GGGGTAGGAA	TGACAAGAAT	TATAAGAGTC	AAATATAGAG	GCTGTGGGCG	AGGCTTCAAT	GATAGGTCAC	ATCTCAGC
JF750647.1|Canis_lupus_familiaris_Eurasier	GGGGTAGGAA	TGACAAGAAT	TATAAGAGTC	AAATATAGAG	GCTGTGGGCG	AGGCTTCAAT	GATAGGTCAC	ATCTCAGC
JF750648.1|Canis_lupus_familiaris_Polish_Lowland_Sheepdog	GGGGTAGGAA	TGACAAGAAT	TATAAGAGTC	AAATATAGAG	GCTGTGGGCG	AGGCTTCAAT	GATAGGTCAC	ATCTCAGC
JF750649.1|Canis_lupus_familiaris_Rottweiler	GGGGTAGGAA	TGACAAGAAT	TATAAGAGTC	AAATATAGAG	GCTGTGGGCG	AGGCTTCAAT	GATAGGTCAC	ATCTCAGC
JF750650.1|Canis_lupus_familiaris_Smalandsstrovare	GGGGTAGGAA	TGACAAGAAT	TATAAGAGTC	AAATATAGAG	GCTGTGGGCG	AGGCTTCAAT	GATAGGTCAC	ATCTCAGC
JF750651.1|Canis_lupus_familiaris_Swedish_Elkhound	GGGGTAGGAA	TGACAAGAAT	TATAAGAGTC	AAATATAGAG	GCTGTGGGCG	AGGCTTCAAT	GATAGGTCAC	ATCTCAGC
JF750652.1|Canis_lupus_familiaris_Nova_Scotia_Duck_Tolling_Retriever	GGGGTAGGAA	TGACAAGAAT	TATAAGAGTC	AAATATAGAG	GCTGTGGGCG	AGGCTTCAAT	GATAGGTCAC	ATCTCAGC
JF750653.1|Canis_lupus_isolate_ref21	GGGGTAGGAA	TGACAAGAAT	TATAAGAGTC	AAATATAGAG	GCTGTGGGCG	AGGCTTCAAT	GATAGGTCAC	ATCTCAGC
JF750654.1|Canis_lupus_isolate_ref34	GGGGTAGGAA	TGACAAGAAT	TATAAGAGTC	AAATATAGAG	GCTGTGGGCG	AGGCTTCAAT	GATAGGTCAC	ATCTCAGC
JF750655.1|Vulpes_vulpes	GGGGTAGGAA	TGACAAGAAT	TATAAGAGTC	AAATATAGAG	GCTGTGGGCG	AGGCTTCAAT	GATAGGTCAC	ATCTCAGC
JF750656.1|Speothos_venaticus	GGGGTAGGAA	TGACAAGAAT	TATAAGAGTC	AAATATAGAG	GCTGTGGGCG	AGGCTTCAAT	GATAGGTCAC	ATCTCAGC
JF750657.1|Lycaon_pictus	GGGGTAGGAA	TGACAAGAAT	TAT--GAGTC	AAATATAGAG	GCTGTGGGCG	AGGCTTCAAT	GATAGGTCAC	ATCTCAGC
JF750658.1|Canis_mesomelas	GGGGTAGGAA	TGACAAGAAT	TATAAGAGTC	AAATATAGAG	GCTGTGGGCG	AGGCTTCAAT	GATAGGTCAC	ATCTCAGC
JF750659.1|Canis_aureus	GGGGTAGGAA	TGACAAGAAT	TATAAGAGTC	AAATATAGAG	GCTGTGGGCG	AGGCTTCAAT	GATAGGTCAC	ATCTCAGC
Island_Fox|Urocyon littoralis	GGGGTAGGAA	TGACAAGAAT	TATAAGAGTC	AAATATAGAG	GCTGTGGGCA	AGGCTTCAAT	GATAGGTCAC	ATCTCAGC
Culpeo|Lycalopex culpaeus	GGGGTAGGAA	TGACAAGAAT	TATAAGAGTC	AAATATAGAG	GCTGTGGGCG	AGGCTTCAAT	GATAGGTCAC	ATCTCAGC

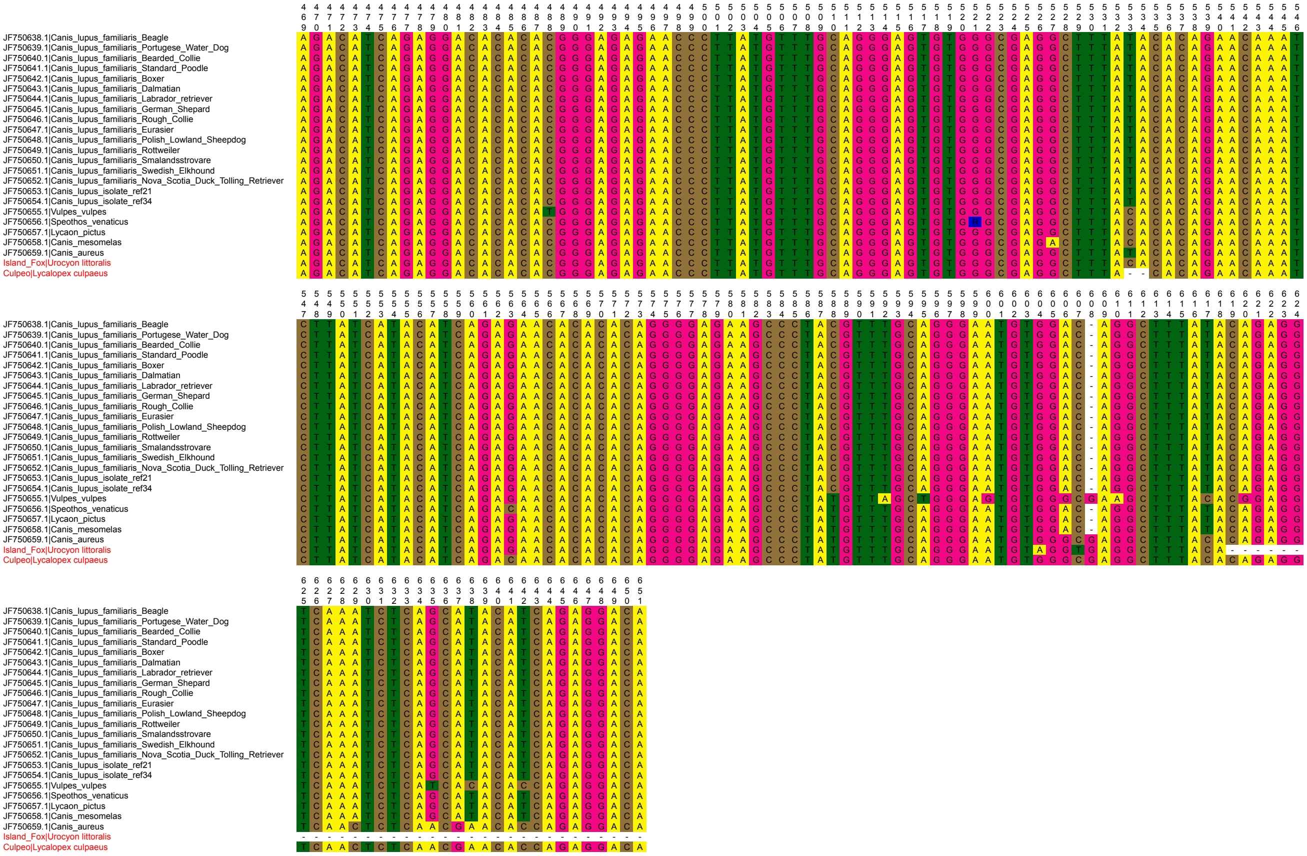
JF750638.1|Canis_lupus_familiaris_Beagle
JF750639.1|Canis_lupus_familiaris_Portugese_Water_Dog
JF750640.1|Canis_lupus_familiaris_Bearded_Collie
JF750641.1|Canis_lupus_familiaris_Standard_Poodle
JF750642.1|Canis_lupus_familiaris_Boxer
JF750643.1|Canis_lupus_familiaris_Dalmatian
JF750644.1|Canis_lupus_familiaris_Labrador_retriever
JF750645.1|Canis_lupus_familiaris_German_Shepard
JF750646.1|Canis_lupus_familiaris_Rough_Collie
JF750647.1|Canis_lupus_familiaris_Eurasier
JF750648.1|Canis_lupus_familiaris_Polish_Lowland_Sheepdog
JF750649.1|Canis_lupus_familiaris_Rottweiler
JF750650.1|Canis_lupus_familiaris_Smalandsstrovare
JF750651.1|Canis_lupus_familiaris_Swedish_Elkhound
JF750652.1|Canis_lupus_familiaris_Nova_Scotia_Duck_Tolling_Retriever
JF750653.1|Canis_lupus_isolate_ref21
JF750654.1|Canis_lupus_isolate_ref34
JF750655.1|Vulpes_vulpes
JF750656.1|Speothos_venaticus
JF750657.1|Lycaon_pictus
JF750658.1|Canis_mesomelas
JF750659.1|Canis_aureus
Island_Fox|Urocyon littoralis
Culpeo|Lycalopex culpaeus
