# Supplementary material for: Genetic Recombination Is Targeted towards Gene Promoter Regions in Dogs
Source: PLoS Genet. 2013 Dec 12;9(12):e1003984. doi: 10.1371/journal.pgen.1003984 (PMC3861134; doi:10.1371/journal.pgen.1003984)
Supplement: Table S5 — PRDM9 exon 7 Amino Acid sequences. Data is as for Table S4, but translated to amino acid sequence. The frameshift mutations in the Andean Fox (Culpeo) can be seen at positions 14 and 178, with the stop codon at position 44. The Island Fox stop codon can be seen at position 203. (PDF) [file pgen.1003984.s018.pdf]

**Table S5: PRDM9 exon 7 Amino Acid sequences**

| | 1 | 2 | 3 | 4 | 5 | 6 | 7 | 8 | 9 | 10 | 11 | 12 | 13 | 14 | 15 | 16 | 17 | 18 | 19 | 20 | 21 | 22 | 23 | 24 | 25 | 26 | 27 | 28 | 29 | 30 | 31 | 32 | 33 | 34 | 35 | 36 | 37 | 38 | 39 | 40 | 41 | 42 | 43 | 44 | 45 | 46 | 47 | 48 | 49 | 50 | 51 | 52 | 53 | 54 | 55 | 56 | 57 | 58 | 59 | 60 | 61 | 62 | 63 | 64 | 65 | 66 | 67 | 68 | 69 | 70 | 71 | 72 | 73 | 74 | 75 | 76 | 77 | 78 | 79 | 80 |
|---|---|---|---|---|---|---|---|---|---|---|---|---|---|---|---|---|---|---|---|---|---|---|---|---|---|---|---|---|---|---|---|---|---|---|---|---|---|---|---|---|---|---|---|---|---|---|---|---|---|---|---|---|---|---|---|---|---|---|---|---|---|---|---|---|---|---|---|---|---|---|---|---|---|---|---|---|---|---|---|---|
| JF750638.1\|Canis_lupus_familiaris_Beagle | P | N | P | E | I | H | P | C | P | S | C | S | L | - | F | S | S | Q | K | F | L | S | Q | H | L | E | H | ? | H | P | S | Q | I | L | P | R | I | S | V | R | E | H | F | R | P | K | D | P | C | P | G | C | Q | N | Q | Q | Q | Q | Q | H | S | D | P | Q | R | W | N | D | R | A | K | G | Q | E | G | K | E | R | F | K |
| JF750639.1\|Canis_lupus_familiaris_Portugese_Water_Dog | P | N | P | E | I | H | P | C | P | S | C | S | L | - | F | S | S | Q | K | F | L | S | Q | H | L | E | H | N | H | P | S | Q | I | L | P | R | I | S | V | R | E | H | F | R | P | K | D | P | C | P | G | C | Q | N | Q | Q | Q | Q | Q | H | S | D | P | Q | R | W | N | D | R | A | K | G | Q | E | G | K | E | R | F | K |
| JF750640.1\|Canis_lupus_familiaris_Bearded_Collie | P | N | P | E | I | H | P | C | P | S | C | S | L | - | F | S | S | Q | K | F | L | S | Q | H | L | E | H | N | H | P | S | Q | I | L | P | R | I | S | V | R | E | H | F | R | P | K | D | P | C | P | G | C | Q | N | Q | Q | Q | Q | Q | H | S | D | P | Q | R | W | N | D | R | A | K | G | Q | E | G | K | E | R | F | K |
| JF750641.1\|Canis_lupus_familiaris_Standard_Poodle | P | N | P | E | I | H | P | C | P | S | C | S | L | - | F | S | S | Q | K | F | L | S | Q | H | L | E | H | N | H | P | S | Q | I | L | P | R | I | S | V | R | E | H | F | R | P | K | D | P | C | P | G | C | Q | N | Q | Q | Q | Q | Q | H | S | D | P | Q | R | W | N | D | R | A | K | G | Q | E | G | K | E | R | F | K |
| JF750642.1\|Canis_lupus_familiaris_Boxer | P | N | P | E | I | H | P | C | P | S | C | S | L | - | F | S | S | Q | K | F | L | S | Q | H | L | E | H | N | H | P | S | Q | I | L | P | R | I | S | V | R | E | H | F | R | P | K | D | P | C | P | G | C | Q | N | Q | Q | Q | Q | Q | H | S | D | P | Q | R | W | N | D | R | A | K | G | Q | E | G | K | E | R | F | K |
| JF750643.1\|Canis_lupus_familiaris_Dalmatian | P | N | P | E | I | H | P | C | P | S | C | S | L | - | F | S | S | Q | K | F | L | S | Q | H | L | E | H | N | H | P | S | Q | I | L | P | R | I | S | V | R | E | H | F | R | P | K | D | P | C | P | G | C | Q | N | Q | Q | Q | Q | Q | H | S | D | P | Q | R | W | N | D | R | A | K | G | Q | E | G | K | E | R | F | K |
| JF750644.1\|Canis_lupus_familiaris_Labrador_retriever | P | N | P | E | I | H | P | C | P | S | C | S | L | - | F | S | S | Q | K | F | L | S | Q | H | L | E | H | N | H | P | S | Q | I | L | P | R | I | S | V | R | E | H | F | R | P | K | D | P | C | P | G | C | Q | N | Q | Q | Q | Q | Q | H | S | D | P | Q | R | W | N | D | R | A | K | G | Q | E | G | K | E | R | F | K |
| JF750645.1\|Canis_lupus_familiaris_German_Shepard | P | N | P | E | I | H | P | C | P | S | C | S | L | - | F | S | S | Q | K | F | L | S | Q | H | L | E | H | N | H | P | S | Q | I | L | P | R | I | S | V | R | E | H | F | R | P | K | D | P | C | P | G | C | Q | N | Q | Q | Q | Q | Q | H | S | D | P | Q | R | W | N | D | R | A | K | G | Q | E | G | K | E | R | F | K |
| JF750646.1\|Canis_lupus_familiaris_Rough_Collie | P | N | P | E | I | H | P | C | P | S | C | S | L | - | F | S | S | Q | K | F | L | S | Q | H | L | E | H | N | H | P | S | Q | I | L | P | R | I | S | V | R | E | H | F | R | P | K | D | P | C | P | G | C | Q | N | Q | Q | Q | Q | Q | H | S | D | P | Q | R | W | N | D | R | A | K | G | Q | E | G | K | E | R | F | K |
| JF750647.1\|Canis_lupus_familiaris_Eurasier | P | N | P | E | I | H | P | C | P | S | C | S | L | - | F | S | S | Q | K | F | L | S | Q | H | L | E | H | N | H | P | S | Q | I | L | P | R | I | S | V | R | E | H | F | R | P | K | D | P | C | P | G | C | Q | N | Q | Q | Q | Q | Q | H | S | D | P | Q | R | W | N | D | R | A | K | G | Q | E | G | K | E | R | F | K |
| JF750648.1\|Canis_lupus_familiaris_Polish_Lowland_Sheepdog | P | N | P | E | I | ? | ? | ? | P | S | C | S | L | - | F | S | S | Q | K | F | L | S | Q | H | L | E | H | N | H | P | S | Q | I | L | P | R | I | S | V | R | E | H | F | R | P | K | D | P | C | P | G | C | Q | N | Q | Q | Q | Q | Q | H | S | D | P | Q | R | W | N | D | R | A | K | G | Q | E | G | K | E | R | F | K |
| JF750649.1\|Canis_lupus_familiaris_Rottweiler | P | N | P | E | I | H | P | C | P | S | C | S | L | - | F | S | S | Q | K | F | L | S | Q | H | L | E | H | ? | H | P | S | Q | I | L | P | R | I | S | V | R | E | H | F | R | P | K | D | P | C | P | G | C | Q | N | Q | Q | Q | Q | Q | H | S | D | P | Q | R | W | N | D | R | A | K | G | Q | E | G | K | E | R | F | K |
| JF750650.1\|Canis_lupus_familiaris_Smalandsstrovare | P | N | P | E | I | H | P | C | P | S | C | S | L | - | F | S | S | Q | K | F | L | S | ? | H | L | E | H | N | H | P | S | Q | I | L | P | R | I | S | V | R | E | H | F | R | P | K | D | P | C | P | G | C | Q | N | Q | Q | Q | Q | Q | H | S | D | P | Q | R | W | N | D | R | A | K | G | Q | E | G | K | E | R | F | K |
| JF750651.1\|Canis_lupus_familiaris_Swedish_Elkhound | P | N | P | E | I | ? | ? | ? | P | S | C | S | L | - | F | S | S | Q | K | F | L | S | Q | H | L | E | H | ? | H | P | S | Q | I | L | P | R | I | S | V | R | E | H | F | R | P | K | D | P | C | P | G | C | Q | N | Q | Q | Q | Q | Q | H | S | D | P | Q | R | W | N | D | R | A | K | G | Q | E | G | K | E | R | F | K |
| JF750652.1\|Canis_lupus_familiaris_Nova_Scotia_Duck_Tolling_Retriever | P | N | P | E | I | H | P | C | P | S | C | S | L | - | F | S | S | Q | K | F | L | S | Q | H | L | E | H | N | H | P | S | Q | I | L | P | R | I | S | V | R | E | H | F | R | P | K | D | P | C | P | G | C | Q | N | Q | Q | Q | Q | Q | H | S | D | P | Q | R | W | N | D | R | A | K | G | Q | E | G | K | E | R | F | K |
| JF750653.1\|Canis_lupus_isolate_ref21 | P | N | P | E | I | H | P | C | P | S | C | S | L | - | F | S | S | Q | K | F | L | S | Q | H | L | E | H | N | H | P | S | Q | I | L | P | R | I | S | V | R | E | H | F | R | P | K | D | P | C | P | G | C | Q | N | Q | Q | Q | Q | Q | H | S | D | P | Q | R | W | N | D | R | A | K | G | Q | E | G | K | E | R | F | K |
| JF750654.1\|Canis_lupus_isolate_ref34 | P | N | P | E | I | H | P | C | P | S | C | S | L | - | F | S | S | Q | K | F | L | S | Q | H | L | E | H | N | H | P | S | Q | I | L | P | R | I | S | V | R | E | H | F | R | P | K | D | P | C | P | G | C | Q | N | Q | Q | Q | Q | Q | H | S | D | P | Q | R | W | N | D | R | A | K | G | Q | E | G | K | E | R | F | K |
| JF750655.1\|Vulpes_vulpes | P | N | P | E | I | Y | P | C | P | S | C | S | L | S | F | S | S | Q | K | F | L | S | Q | H | L | E | H | N | H | P | S | Q | I | L | P | R | I | S | I | R | E | H | F | Q | P | K | D | P | C | P | G | C | Q | N | Q | Q | Q | Q | Q | H | S | D | P | Q | C | W | N | D | R | A | K | G | Q | E | G | K | E | R | F | K |
| JF750656.1\|Speothos_venaticus | P | N | P | E | I | H | P | C | P | S | C | S | L | - | F | S | S | Q | K | F | L | S | Q | H | L | E | H | N | H | P | S | Q | I | L | P | * | I | S | V | R | E | H | F | R | P | K | D | P | C | P | G | C | Q | N | Q | Q | Q | Q | Q | H | S | D | P | Q | R | W | N | D | R | A | K | G | Q | E | G | K | E | R | F | K |
| JF750657.1\|Lycaon_pictus | P | N | P | E | I | H | P | C | P | S | C | S | L | - | F | S | S | Q | K | F | L | S | Q | H | L | E | H | N | H | P | S | Q | I | L | P | R | I | S | V | R | E | H | F | R | P | K | D | P | C | P | G | C | Q | N | Q | Q | Q | Q | Q | H | S | D | P | Q | R | W | S | D | R | A | K | G | Q | E | G | K | E | R | F | K |
| JF750658.1\|Canis_mesomelas | P | N | P | E | I | H | P | C | P | S | C | S | L | - | F | S | S | Q | K | F | L | S | Q | H | L | E | H | N | H | P | S | Q | I | L | P | Q | I | S | V | R | E | H | F | R | P | K | D | P | C | P | G | C | Q | N | Q | Q | Q | Q | Q | H | S | D | P | Q | R | W | N | D | R | A | K | G | Q | E | G | K | E | R | F | K |
| JF750659.1\|Canis_aureus | P | N | P | E | I | H | P | C | P | S | C | S | L | - | F | S | S | Q | K | F | L | S | Q | H | L | E | H | N | H | P | S | Q | I | L | P | R | I | S | V | R | E | H | F | R | P | K | D | P | C | P | G | C | Q | N | Q | Q | Q | Q | Q | H | S | D | P | Q | R | W | N | D | R | A | K | G | Q | E | G | K | E | R | F | K |
| Island_Fox\|Urocyon littoralis | ? | N | P | E | I | H | P | C | P | S | C | S | L | A | F | S | S | Q | K | F | L | S | Q | H | L | E | H | N | H | P | ? | Q | I | L | P | R | I | S | V | R | E | H | F | R | P | K | D | P | C | P | G | C | Q | N | Q | Q | Q | Q | Q | H | S | D | P | Q | R | W | N | D | R | A | K | G | Q | E | G | K | E | R | F | K |
| Culpeo\|Lycalopex culpaeus | P | N | P | E | I | H | P | C | P | S | C | S | L | - | F | S | S | Q | K | F | L | S | Q | H | L | E | H | N | H | P | S | Q | I | L | P | R | I | S | V | R | E | H | F | * | P | K | D | P | C | P | G | C | Q | N | Q | Q | Q | Q | Q | H | S | D | P | Q | R | W | N | D | R | A | K | G | Q | E | G | K | E | R | F | K |

| | 81 | 82 | 83 | 84 | 85 | 86 | 87 | 88 | 89 | 90 | 91 | 92 | 93 | 94 | 95 | 96 | 97 | 98 | 99 | 100 | 101 | 102 | 103 | 104 | 105 | 106 | 107 | 108 | 109 | 110 | 111 | 112 | 113 | 114 | 115 | 116 | 117 | 118 | 119 | 120 | 121 | 122 | 123 | 124 | 125 | 126 | 127 | 128 | 129 | 130 | 131 | 132 | 133 | 134 | 135 | 136 | 137 | 138 | 139 | 140 | 141 | 142 | 143 | 144 | 145 | 146 | 147 | 148 | 149 | 150 | 151 | 152 | 153 | 154 | 155 | 156 | 157 | 158 | 159 | 160 |
|---|---|---|---|---|---|---|---|---|---|---|---|---|---|---|---|---|---|---|---|---|---|---|---|---|---|---|---|---|---|---|---|---|---|---|---|---|---|---|---|---|---|---|---|---|---|---|---|---|---|---|---|---|---|---|---|---|---|---|---|---|---|---|---|---|---|---|---|---|---|---|---|---|---|---|---|---|---|---|---|---|
| JF750638.1\|Canis_lupus_familiaris_Beagle | P | L | P | K | S | I | R | Q | R | R | I | S | R | A | F | S | T | P | C | K | G | Q | T | T | C | E | G | I | V | K | E | E | P | S | A | G | S | Q | K | L | N | P | E | D | T | G | K | L | F | K | G | V | G | M | T | R | I | I | R | V | K | Y | R | G | C | G | R | G | F | N | D | R | S | H | L | S | R | H | Q | R |
| JF750639.1\|Canis_lupus_familiaris_Portugese_Water_Dog | P | L | P | K | S | I | R | Q | R | R | I | S | R | A | F | S | T | P | C | K | G | Q | T | T | C | E | G | I | V | K | E | E | P | S | A | G | S | Q | K | L | N | P | E | D | T | G | K | L | F | K | G | V | G | M | T | R | I | I | R | V | K | Y | R | G | C | G | R | G | F | N | D | R | S | H | L | S | R | H | Q | R |
| JF750640.1\|Canis_lupus_familiaris_Bearded_Collie | P | L | P | K | S | I | R | Q | R | R | I | S | R | A | F | S | T | P | C | K | G | Q | T | T | C | E | G | I | V | K | E | E | P | S | A | G | S | Q | K | L | N | P | E | D | T | G | K | L | F | K | G | V | G | M | T | R | I | I | R | V | K | Y | R | G | C | G | R | G | F | N | D | R | S | H | L | S | R | H | Q | R |
| JF750641.1\|Canis_lupus_familiaris_Standard_Poodle | P | L | P | K | S | I | R | Q | R | R | I | S | R | A | F | S | T | P | C | K | G | Q | T | T | C | E | G | I | V | K | E | E | P | S | A | G | S | Q | K | L | N | P | E | D | T | G | K | L | F | K | G | V | G | M | T | R | I | I | R | V | K | Y | R | G | C | G | R | G | F | N | D | R | S | H | L | S | R | H | Q | R |
| JF750642.1\|Canis_lupus_familiaris_Boxer | P | L | P | K | S | I | R | Q | R | R | I | S | R | A | F | S | T | P | C | K | G | Q | T | T | C | E | G | I | V | K | E | E | P | S | A | G | S | Q | K | L | N | P | E | D | T | G | K | L | F | K | G | V | G | M | T | R | I | I | R | V | K | Y | R | G | C | G | R | G | F | N | D | R | S | H | L | S | R | H | Q | R |
| JF750643.1\|Canis_lupus_familiaris_Dalmatian | P | L | P | K | S | I | R | Q | R | R | I | S | R | A | F | S | T | P | C | K | G | Q | T | T | C | E | G | I | V | K | E | E | P | S | A | G | S | Q | K | L | N | P | E | D | T | G | K | L | F | K | G | V | G | M | T | R | I | I | R | V | K | Y | R | G | C | G | R | G | F | N | D | R | S | H | L | S | R | H | Q | R |
| JF750644.1\|Canis_lupus_familiaris_Labrador_retriever | P | L | P | K | S | I | R | Q | R | R | I | S | R | A | F | S | T | P | C | K | G | Q | T | T | C | E | G | I | V | K | E | E | P | S | A | G | S | Q | K | L | N | P | E | D | T | G | K | L | F | K | G | V | G | M | T | R | I | I | R | V | K | Y | R | G | C | G | R | G | F | N | D | R | S | H | L | S | R | H | Q | R |
| JF750645.1\|Canis_lupus_familiaris_German_Shepard | P | L | P | K | S | I | R | Q | R | R | I | S | R | A | F | S | T | P | C | K | G | Q | T | T | C | E | G | I | V | K | E | E | P | S | A | G | S | Q | K | L | N | P | E | D | T | G | K | L | F | K | G | V | G | M | T | R | I | I | R | V | K | Y | R | G | C | G | R | G | F | N | D | R | S | H | L | S | R | H | Q | R |
| JF750646.1\|Canis_lupus_familiaris_Rough_Collie | P | L | P | K | S | I | R | Q | R | R | I | S | R | A | F | S | T | P | C | K | G | Q | T | T | C | E | G | I | V | K | E | E | P | S | A | G | S | Q | K | L | N | P | E | D | T | G | K | L | F | K | G | V | G | M | T | R | I | I | R | V | K | Y | R | G | C | G | R | G | F | N | D | R | S | H | L | S | R | H | Q | R |
| JF750647.1\|Canis_lupus_familiaris_Eurasier | P | L | P | K | S | I | R | Q | R | R | I | S | R | A | F | S | T | P | C | K | G | Q | T | T | C | E | G | I | V | K | E | E | P | S | A | G | S | Q | K | L | N | P | E | D | T | G | K | L | F | K | G | V | G | M | T | R | I | I | R | V | K | Y | R | G | C | G | R | G | F | N | D | R | S | H | L | S | R | H | Q | R |
| JF750648.1\|Canis_lupus_familiaris_Polish_Lowland_Sheepdog | P | L | P | K | S | I | R | Q | R | R | I | S | R | A | F | S | T | P | C | K | G | Q | T | T | C | E | G | I | V | K | E | E | P | S | A | G | S | Q | K | L | N | P | E | D | T | G | K | L | F | K | G | V | G | M | T | R | I | I | R | V | K | Y | R | G | C | G | R | G | F | N | D | R | S | H | L | S | R | H | Q | R |
| JF750649.1\|Canis_lupus_familiaris_Rottweiler | P | L | P | K | S | I | R | Q | R | R | I | S | R | A | F | S | T | P | C | K | G | Q | T | T | C | E | G | I | V | K | E | E | P | S | A | G | S | Q | K | L | N | P | E | D | T | G | K | L | F | K | G | V | G | M | T | R | I | I | R | V | K | Y | R | G | C | G | R | G | F | N | D | R | S | H | L | S | R | H | Q | R |
| JF750650.1\|Canis_lupus_familiaris_Smalandsstrovare | P | L | P | K | S | I | R | Q | R | R | I | S | R | A | F | S | T | P | C | K | G | Q | T | T | C | E | G | I | V | K | E | E | P | S | A | G | S | Q | K | L | N | P | E | D | T | G | K | L | F | K | G | V | G | M | T | R | I | I | R | V | K | Y | R | G | C | G | R | G | F | N | D | R | S | H | L | S | R | H | Q | R |
| JF750651.1\|Canis_lupus_familiaris_Swedish_Elkhound | P | L | P | K | S | I | R | Q | R | R | I | S | R | A | F | S | T | P | C | K | G | Q | T | T | C | E | G | I | V | K | E | E | P | S | A | G | S | Q | K | L | N | P | E | D | T | G | K | L | F | K | G | V | G | M | T | R | I | I | R | V | K | Y | R | G | C | G | R | G | F | N | D | R | S | H | L | S | R | H | Q | R |
| JF750652.1\|Canis_lupus_familiaris_Nova_Scotia_Duck_Tolling_Retriever | P | L | P | K | S | I | R | Q | R | R | I | S | R | A | F | S | T | P | C | K | G | Q | T | T | C | E | G | I | V | K | E | E | P | S | A | G | S | Q | K | L | N | P | E | D | T | G | K | L | F | K | G | V | G | M | T | R | I | I | R | V | K | Y | R | G | C | G | R | G | F | N | D | R | S | H | L | S | R | H | Q | R |
| JF750653.1\|Canis_lupus_isolate_ref21 | P | L | P | K | S | I | R | Q | R | R | I | S | R | A | F | S | T | P | C | K | G | Q | T | T | C | E | G | I | V | K | E | E | P | S | A | G | S | Q | K | L | N | P | E | D | T | G | K | L | F | K | G | V | G | M | T | R | I | I | R | V | K | Y | R | G | C | G | R | G | F | N | D | R | S | H | L | S | R | H | Q | R |
| JF750654.1\|Canis_lupus_isolate_ref34 | P | L | P | K | S | I | R | Q | R | R | I | S | R | A | F | S | T | P | C | K | G | Q | T | T | C | E | G | I | V | K | E | E | P | S | A | G | S | Q | K | L | N | P | E | D | T | G | K | L | F | K | G | V | G | M | T | R | I | I | R | V | K | Y | R | G | C | G | R | G | F | N | D | R | S | H | L | S | R | H | Q | R |
| JF750655.1\|Vulpes_vulpes | P | L | P | K | - | I | R | Q | R | K | I | S | R | A | F | S | T | P | C | K | G | Q | T | T | C | E | G | I | V | K | E | E | P | S | A | G | S | Q | K | L | N | P | E | D | T | G | K | L | F | K | G | V | G | M | T | R | I | I | R | V | K | Y | R | G | C | G | R | G | F | N | D | R | S | H | L | S | R | H | Q | R |
| JF750656.1\|Speothos_venaticus | P | L | P | K | R | I | R | Q | R | R | I | S | R | A | F | S | T | P | C | K | G | Q | T | T | C | E | G | I | V | K | E | E | P | S | A | S | S | Q | K | L | N | P | E | ? | T | G | K | L | F | K | G | V | G | M | T | R | I | I | R | V | K | Y | R | G | C | G | R | G | F | N | D | R | S | H | L | S | R | H | Q | R |
| JF750657.1\|Lycaon_pictus | P | L | P | K | S | I | R | Q | R | R | I | S | R | A | F | S | T | P | C | K | G | Q | T | T | C | E | G | I | V | K | E | E | P | S | A | G | S | Q | K | L | N | P | E | D | T | G | K | L | F | K | G | V | G | M | T | R | I | - | - | V | K | Y | R | G | C | G | R | G | F | N | D | R | S | H | L | S | R | H | Q | R |
| JF750658.1\|Canis_mesomelas | P | L | P | K | S | I | R | Q | R | R | I | S | R | A | F | S | T | P | C | K | G | Q | T | T | C | E | G | I | V | K | E | E | P | S | A | G | S | Q | K | L | N | P | E | D | T | G | K | L | F | K | G | V | G | M | T | R | I | I | R | V | K | Y | R | G | C | G | R | G | F | N | D | R | S | H | L | S | R | H | Q | R |
| JF750659.1\|Canis_aureus | P | L | P | K | S | I | R | Q | R | R | I | S | R | A | F | S | T | P | C | K | G | Q | T | T | C | E | G | I | V | K | E | E | P | S | A | G | S | Q | K | L | N | P | E | D | T | G | K | L | F | K | G | V | G | M | T | R | I | I | R | V | K | Y | R | G | C | G | R | G | F | N | D | R | S | H | L | S | R | H | Q | R |
| Island_Fox\|Urocyon littoralis | P | L | P | K | S | I | R | Q | R | R | I | S | R | A | F | S | T | P | C | K | G | Q | T | T | C | E | G | I | V | K | E | E | P | S | T | G | S | Q | K | L | N | P | E | D | T | G | K | L | F | K | G | V | G | M | T | R | I | I | R | V | K | Y | R | G | C | G | Q | G | F | N | D | R | S | H | L | S | R | H | Q | R |
| Culpeo\|Lycalopex culpaeus | P | L | P | K | R | I | R | Q | R | R | I | S | R | A | F | S | T | P | C | K | G | Q | T | T | C | E | G | I | V | K | E | E | P | S | A | G | S | Q | K | L | N | P | E | D | T | G | K | L | F | K | G | V | G | M | T | R | I | I | R | V | K | Y | R | G | C | G | R | G | F | N | D | R | S | H | L | S | R | H | Q | R |

| | 161 | 162 | 163 | 164 | 165 | 166 | 167 | 168 | 169 | 170 | 171 | 172 | 173 | 174 | 175 | 176 | 177 | 178 | 179 | 180 | 181 | 182 | 183 | 184 | 185 | 186 | 187 | 188 | 189 | 190 | 191 | 192 | 193 | 194 | 195 | 196 | 197 | 198 | 199 | 200 | 201 | 202 | 203 | 204 | 205 | 206 | 207 | 208 | 209 | 210 | 211 | 212 | 213 | 214 | 215 | 216 | 217 |
|---|---|---|---|---|---|---|---|---|---|---|---|---|---|---|---|---|---|---|---|---|---|---|---|---|---|---|---|---|---|---|---|---|---|---|---|---|---|---|---|---|---|---|---|---|---|---|---|---|---|---|---|---|---|---|---|---|---|
| JF750638.1\|Canis_lupus_familiaris_Beagle | T | H | T | G | E | N | P | Y | V | C | R | E | C | G | R | G | F | I | H | R | T | N | L | I | I | H | Q | R | T | H | T | G | E | K | P | Y | V | C | R | E | C | G | - | G | F | I | Q | R | S | N | L | S | I | H | Q | R | T |
| JF750639.1\|Canis_lupus_familiaris_Portugese_Water_Dog | T | H | T | G | E | N | P | Y | V | C | R | E | C | G | R | G | F | I | H | R | T | N | L | I | I | H | Q | R | T | H | T | G | E | K | P | Y | V | C | R | E | C | G | - | G | F | I | Q | R | S | N | L | S | I | H | Q | R | T |
| JF750640.1\|Canis_lupus_familiaris_Bearded_Collie | T | H | T | G | E | N | P | Y | V | C | R | E | C | G | R | G | F | I | H | R | T | N | L | I | I | H | Q | R | T | H | T | G | E | K | P | Y | V | C | R | E | C | G | - | G | F | I | Q | R | S | N | L | S | I | H | Q | R | T |
| JF750641.1\|Canis_lupus_familiaris_Standard_Poodle | T | H | T | G | E | N | P | Y | V | C | R | E | C | G | R | G | F | I | H | R | T | N | L | I | I | H | Q | R | T | H | T | G | E | K | P | Y | V | C | R | E | C | G | - | G | F | I | Q | R | S | N | L | S | I | H | Q | R | T |
| JF750642.1\|Canis_lupus_familiaris_Boxer | T | H | T | G | E | N | P | Y | V | C | R | E | C | G | R | G | F | I | H | R | T | N | L | I | I | H | Q | R | T | H | T | G | E | K | P | Y | V | C | R | E | C | G | - | G | F | I | Q | R | S | N | L | S | I | H | Q | R | T |
| JF750643.1\|Canis_lupus_familiaris_Dalmatian | T | H | T | G | E | N | P | Y | V | C | R | E | C | G | R | G | F | I | H | R | T | N | L | I | I | H | Q | R | T | H | T | G | E | K | P | Y | V | C | R | E | C | G | - | G | F | I | Q | R | S | N | L | S | I | H | Q | R | T |
| JF750644.1\|Canis_lupus_familiaris_Labrador_retriever | T | H | T | G | E | N | P | Y | V | C | R | E | C | G | R | G | F | I | H | R | T | N | L | I | I | H | Q | R | T | H | T | G | E | K | P | Y | V | C | R | E | C | G | - | G | F | I | Q | R | S | N | L | S | I | H | Q | R | T |
| JF750645.1\|Canis_lupus_familiaris_German_Shepard | T | H | T | G | E | N | P | Y | V | C | R | E | C | G | R | G | F | I | H | R | T | N | L | I | I | H | Q | R | T | H | T | G | E | K | P | Y | V | C | R | E | C | G | - | G | F | I | Q | R | S | N | L | S | I | H | Q | R | T |
| JF750646.1\|Canis_lupus_familiaris_Rough_Collie | T | H | T | G | E | N | P | Y | V | C | R | E | C | G | R | G | F | I | H | R | T | N | L | I | I | H | Q | R | T | H | T | G | E | K | P | Y | V | C | R | E | C | G | - | G | F | I | Q | R | S | N | L | S | I | H | Q | R | T |
| JF750647.1\|Canis_lupus_familiaris_Eurasier | T | H | T | G | E | N | P | Y | V | C | R | E | C | G | R | G | F | I | H | R | T | N | L | I | I | H | Q | R | T | H | T | G | E | K | P | Y | V | C | R | E | C | G | - | G | F | I | Q | R | S | N | L | S | I | H | Q | R | T |
| JF750648.1\|Canis_lupus_familiaris_Polish_Lowland_Sheepdog | T | H | T | G | E | N | P | Y | V | C | R | E | C | G | R | G | F | I | H | R | T | N | L | I | I | H | Q | R | T | H | T | G | E | K | P | Y | V | C | R | E | C | G | - | G | F | I | Q | R | S | N | L | S | I | H | Q | R | T |
| JF750649.1\|Canis_lupus_familiaris_Rottweiler | T | H | T | G | E | N | P | Y | V | C | R | E | C | G | R | G | F | I | H | R | T | N | L | I | I | H | Q | R | T | H | T | G | E | K | P | Y | V | C | R | E | C | G | - | G | F | I | Q | R | S | N | L | S | I | H | Q | R | T |
| JF750650.1\|Canis_lupus_familiaris_Smalandsstrovare | T | H | T | G | E | N | P | Y | V | C | R | E | C | G | R | G | F | I | H | R | T | N | L | I | I | H | Q | R | T | H | T | G | E | K | P | Y | V | C | R | E | C | G | - | G | F | I | Q | R | S | N | L | S | I | H | Q | R | T |
| JF750651.1\|Canis_lupus_familiaris_Swedish_Elkhound | T | H | T | G | E | N | P | Y | V | C | R | E | C | G | R | G | F | I | H | R | T | N | L | I | I | H | Q | R | T | H | T | G | E | K | P | Y | V | C | R | E | C | G | - | G | F | I | Q | R | S | N | L | S | I | H | Q | R | T |
| JF750652.1\|Canis_lupus_familiaris_Nova_Scotia_Duck_Tolling_Retriever | T | H | T | G | E | N | P | Y | V | C | R | E | C | G | R | G | F | I | H | R | T | N | L | I | I | H | Q | R | T | H | T | G | E | K | P | Y | V | C | R | E | C | G | - | G | F | I | Q | R | S | N | L | S | I | H | Q | R | T |
| JF750653.1\|Canis_lupus_isolate_ref21 | T | H | T | G | E | N | P | Y | V | C | R | E | C | G | R | G | F | I | H | R | T | N | L | I | I | H | Q | R | T | H | T | G | E | K | P | Y | V | C | R | E | C | G | - | G | F | I | Q | R | S | N | L | S | I | H | Q | R | T |
| JF750654.1\|Canis_lupus_isolate_ref34 | T | H | T | G | E | N | P | Y | V | C | R | E | C | G | R | G | F | I | H | R | T | N | L | I | I | H | Q | R | T | H | T | G | E | K | P | Y | V | C | R | E | C | G | - | G | F | I | Q | R | S | N | L | S | I | H | Q | R | T |
| JF750655.1\|Vulpes_vulpes | T | H | M | G | E | N | P | Y | V | C | R | E | C | G | R | G | F | T | H | R | T | N | L | I | I | H | Q | R | T | H | T | G | E | K | P | Y | V | S | W | E | C | G | R | S | F | T | R | R | S | N | L | I | T | H | Q | R | T |
| JF750656.1\|Speothos_venaticus | T | H | T | G | E | N | P | Y | V | C | R | E | C | ? | R | G | F | T | H | R | T | N | L | I | I | H | Q | T | T | H | T | G | E | K | P | Y | V | C | R | E | C | G | - | G | F | I | Q | R | S | N | L | S | I | H | Q | R | T |
| JF750657.1\|Lycaon_pictus | T | H | T | G | E | N | P | Y | V | C | R | E | C | G | R | G | F | T | H | R | T | N | L | I | I | H | Q | R | T | H | T | G | E | K | P | Y | V | C | R | E | C | G | - | G | F | I | Q | R | S | N | L | S | I | H | Q | R | T |
| JF750658.1\|Canis_mesomelas | T | H | T | G | E | N | P | Y | V | C | R | E | C | G | R | D | F | T | H | R | T | N | L | I | I | H | Q | R | T | H | T | G | E | K | P | Y | V | C | R | E | C | G | - | G | F | I | Q | R | S | N | L | S | I | H | Q | R | T |
| JF750659.1\|Canis_aureus | T | H | T | G | E | N | P | Y | V | C | R | E | C | G | R | G | F | I | H | R | T | N | L | I | I | H | Q | R | T | H | T | G | E | K | P | Y | V | C | R | E | C | G | R | G | F | T | Q | R | S | T | L | N | E | H | Q | R | T |
| Island_Fox\|Urocyon littoralis | T | H | T | G | E | N | P | Y | V | C | R | E | C | G | R | G | F | T | H | R | T | N | L | I | I | H | Q | R | T | H | T | G | E | K | P | Y | V | C | R | E | C | R | * | G | F | T | - | - | - | - | - | - | - | - | - | - | - |
| Culpeo\|Lycalopex culpaeus | T | H | T | G | E | N | P | Y | V | C | R | E | C | G | R | G | F | - | H | R | T | N | L | I | I | H | Q | T | T | H | T | G | E | K | P | Y | V | C | R | E | C | G | R | G | F | T | Q | R | S | T | L | N | E | H | Q | R | T |
