# Supplementary material for: Genetic Recombination Is Targeted towards Gene Promoter Regions in Dogs
Source: PLoS Genet. 2013 Dec 12;9(12):e1003984. doi: 10.1371/journal.pgen.1003984 (PMC3861134; doi:10.1371/journal.pgen.1003984)
Supplement: Table S6 — Primers used for qPCR validation of ChIP. (PDF) [file pgen.1003984.s019.pdf]

**Table S6: Primers used for qPCR validation of ChIP**

| | |
|---|---|
| SYCE1.F | GTGTGTGGCATAAGAGTTTGTGTAT |
| SYCE1.R | AGATTAAAACAGGCAGGAGGAT |
| MLH1.F | TAGTGACCCAACTTAGTGTTTTCCT |
| MLH1.R | CTTTCTCTCCAGGTTCTTAACCTCT |
| Tex12.F | TATATGCACTCATGTCCCACTTAG |
| Tex12.R | AAACATCTACCCTTTCAGGATACAG |
| Tex26.F | TACTTGCTTGTCAGACTCTAGCA |
| Tex26.R | GGTATCTTTAGAGCCAATCTAGGAC |

All sequences are listed 5’ to 3’
