# Supplementary material for: Genetic Recombination Is Targeted towards Gene Promoter Regions in Dogs
Source: PLoS Genet. 2013 Dec 12;9(12):e1003984. doi: 10.1371/journal.pgen.1003984 (PMC3861134; doi:10.1371/journal.pgen.1003984)
Supplement: Text S1 — Details of supplementary methods and analyses. (PDF) [file pgen.1003984.s020.pdf]

Genetic recombination is targeted towards gene promoter regions in dogs - Supplementary Material

Sample collection

Dogs were sampled between 2007 and 2010 at multiple locations throughout the world (Table S1). We selected approximately 6 samples from various regions for sequencing, focusing mainly on 'free-ranging' village dogs which are generally more genetically diverse due to the absence of breed founder effects and artificial selection [17]. Dogs from China were selected for sequencing in an effort to maximize the geographical spread of the samples (1-2 for most provinces), and some of these are associated with breeds.

DNA was collected from 3-5 ml blood samples under Cornell IACUC #2005-0151, 2007-0076 and 2011-0061. DNA was extracted from a captive female culpeo fox (*Lycalopex culpaeus culpaeus*) from the Cusco region of Peru using a similar procedure.

Sequencing

Paired end libraries were prepared from genomic DNA using the standard Illumina protocols. Briefly, genomic DNA was sheared in a Covaris E210. Sheared DNA was end-repaired, A-tailed, gel-purified and ligated with Illumina's paired end adapters. Libraries were amplified for 8 cycles. Libraries were run for 2 x 101 bp reads on one lane each on a HiSeq 2000. This procedure resulted in between 8X and 12X coverage for each sample.

Mapping and variant calling

The CanFam3.1/canFam3 reference assembly was downloaded from the Broad FTP site [36] in October 2010 and converted to FASTA format. We note that this reference differs from the final CanFam3.1/canFam3 reference released by UCSC due to differences in choices regarding padding between contigs. Sequence reads from 57 dogs were mapped to reference using *bwa* [31]. PCR duplicates were excluded using *Picard* [37], and empirical quality recalibration and realignment around candidate indels was performed using *GATK* [32]. SNP genotype likelihoods were generated by *GATK* and passed to *BEAGLE* [33] for genotype calling and phasing.

The Andean fox sequence was processed in a similar manner, with reads also aligned to the canine reference genome. *GATK* was also used to generate genotype calls at dog variant sites, yielding a 95% call rate with approximately 1% heterozygous genotype calls. These variant calls were used to polarize the ancestral allele for polymorphisms discovered in dog, with 3,296,170 sites being assigned an

ancestral allele with high confidence. In addition, *GATK* was also used to perform SNP discovery in the fox, allowing discovery of 1,287,138 heterozygote polymorphisms in the fox with a SNP quality > 100 and a genotype quality >= 99.

Calculation of variant detection power and genotype accuracy

A total of 48 of the 51 sequenced dogs were genotyped on Illumina CanineHD microarrays [18]. After filter out sites with low call rates (<90%), low duplicate concordance rates across genotyping platforms, or sites at positions which did not uniquely map from canFam2 to canFam3 with liftOver, we estimate that we have >98% power to detect variants with minor allele frequency of 5%, and a nearly 95% power to detect rarer variants. For these variants detected by both genotyping and sequencing approaches, the overall concordance of the genotype calls is 99.1%.

Construction of a genetic map

Prior to estimating the genetic map, we conducted extensive filtering of the variants called in the filtering. In part, this was because past experience has indicated that estimating recombination rates from patterns of LD can be moderately sensitive false-positive variant calls [10]. Specifically, we filtered variants to exclude all indels, and non-biallelic variants. In addition we filtered SNPs with unusual depth of coverage (average coverage across individuals <6X or >12X), or highly diverged from Hardy Weinberg Equilibrium (the number of heterozygote genotypes was < 60% or > 100% than the expectation under HWE). In order to focus on only very high quality variants, we excluded any variant in a homopolymer of length > 3.

The resulting callset consisted of 3,560,506 polymorphic SNPs on the autosomes, 145,431 SNPs on the non-pseudoautosomal X chromosome, and 18,285 SNPs in the pseudoautosome.

The genetic map was estimated using the *LDhat* [15,38] software package, which uses a coalescent-based model to infer historical recombination rates from population genetic data. To estimate the genetic map, we followed a procedure similar to that used previously [10,14,15,22]. Specifically, we split the dataset into 4000 SNP windows with a 200 SNP overlap between windows. Recombination rates were estimated by *LDhat* for each window independently using a block penalty of 5 with 60 million MCMC iterations. A sample was taken from the chain every 40,000 iterations, and the first 500 samples (corresponding to 20 million iterations) were discarded as burn-in.

In addition, preliminary analysis revealed a strong association with CpG-rich regions of the genome. Therefore, all recombination analyses have been repeated having excluded all putative CpG sites (i.e. either SNP allele would result in a CpG site), as well as any SNP within 1kb of a putative CpG island, from the call set to ensure that the resulting observations were robust to any CpG-associated artifacts. We also investigated stronger filters that those described above, including filtering

out sites with low minor allele frequencies or mapping to repetitive elements in the genome, but found them to have a minimal qualitative impact on the final estimates. Likewise, we conducted investigations regarding which dogs to include in the dataset when generating the genetic map. We found that using population subsets of dogs did not qualitatively alter patterns observed in final estimates, and hence we chose to include dogs that are not strictly village dogs as long as their inbreeding was not too severe (F > 50%). Three purebred dogs (a basenji, a Mongolia shepherd, and Perdiguero) were excluded, as were three New Guinea Singing dogs, either due to high inbreeding or due to being outliers in PC1 and PC2 when the genotype data was analyzed using principal component analysis. We have replicated the results described in this paper using more conservative call sets with more selective dog and SNP selection procedures, and have found them to be robust.

LDhat provides estimates of the population recombination rate, $\rho = 4N_e r$. As *LDhat* estimates recombination rates from patterns of linkage disequilibrium (LD), artifactual breakdowns in LD can result in artificially large, and biologically implausible, estimates of recombination [10]. To this end, we set the recombination rate to zero within a region if the estimate of $4N_e r$ between a pair of adjacent SNPs was greater than 100, or if there was a gap in the reference of greater than 100kb in size. Whenever such a region was identified, the recombination rates for the surrounding 100 SNPs (50 SNPs in both directions) were also zeroed out, as these estimates are also likely to unreliable. In total, these filters zeroed out recombination rates in 0.12% of SNP intervals (arising from 2 gaps > 100kb, and 40 distinct regions with a SNP interval $4N_e r$ estimates > 100).

In order to convert the population estimate into a per-generation recombination rate estimate (measured in units of cM/Mb), it is necessary to obtain an estimate of the effective population size, N_e. In order to do this, we used a robust linear regression (without intercept) between the *LDhat* estimates and the experimental estimates obtained from Wong *et al*. [19] in 5 Mb bins. The gradient of the fitted line can be used to obtain an estimate of N_e. The use of robust regression ensures that local deviations in the correlation between human and dog do not overly influence the N_e estimate. Using this method, we estimated of N_e as 31034.71, which we used to scale the *LDhat* estimates accordingly.

Calling hotspots

Recombination hotspots in the dog genome were called in a similar manner as used previously for chimps [10]. Briefly, to assess if observed peaks in the estimated recombination rate estimates represent significant variation over and above the noise in the estimator we used coalescent simulations to assess the significance of recombination peaks detected in the data. The method detects regions of localized elevated recombination rate by comparing a model in which the recombination rate within a small region at the center of a window is equal to the surrounding background recombination rate (the null model) to a model in which the

recombination rate is allowed to take any value (the alternative model). The algorithm uses coalescent simulations to determine the null distribution of a test statistic in the absence of a recombination hotspot, and compares this distribution to the test statistic obtained from the true data. A hotspot is called if the true data test statistic lies in the extreme tail of the empirical null distribution. The test statistic in this case is the composite likelihood used by *LDhat* [15,38].

The hotspot-detection method processes the data in windows of 100kb, testing the central 3kb for the presence of a recombination hotspot. The window is moved 1kb at each iteration. For each window, a maximum likelihood constant background recombination rate is calculated across the 100kb window. Subsequently, a maximum likelihood estimate of the recombination rate was obtained allowing the central 3kb to take vary from the background. The test statistic is taken as (composite) likelihood ratio between the two models. In order to obtain the null distribution of the test statistic, we performed coalescent simulations using a constant recombination rate drawn from an exponential distribution with mean equal to the background rate measured in the real data. We performed 5,000 simulations for each putative hotspot, although the simulations were cut short if there is no evidence of significance after 50 simulations ($p > 0.01$). Simulations were conducted using the neutral, equilibrium coalescent with recombination and assuming the infinite sites model. The number of mutations was conditioned to match the number of SNPs in the real data, and mutations were placed at SNP locations.

Having tested all 3kb windows in the genome, hotspots were called using the following steps. First, all 3kb windows with $p<0.01$ were selected. Adjacent or overlapping windows were merged to provide a list of putative hotspot regions. After merging, we discarded regions that did not contain at least one window with a p-value < 0.001. At this stage, we called 7,677 putative hotspots with mean width of 21,954 bp (10,000 bootstrap 95% C.I. 21,696 – 22,225 bp).

To further localize the called hotspots, we compared the hotspot regions to the recombination rate estimates obtained from *LDhat*. Specifically, the peak rate was taken as the maximum rate within the significant region, and the boundaries of the peak were taken as the Full Width at Half Maximum (FWHM) if this was smaller than the original significant region boundaries. This procedure resulted in a mean hotspot width of 8,259 bp (10,000 bootstrap 95% C.I. 8,066 – 8,467 bp).

For each hotspot, we considered the GC, CpG, and N (missing) base composition of the reference sequence contained with the hotspot. We discarded hotspots if more than 1% of the reference sequence was missing within the full width region, leaving 5,467 hotspots. The mean FWHM width of these hotspots was 7,540 bp, with 4,255 hotspots (78%) localized to within 10kb, and 2,927 (54%) localized to within 5kb.

Identifying hotspot-associated motifs

For each hotspot, we attempted to identify a nearby region showing no evidence for recombination rate elevation (a 'coldspot'), matched for GC content and SNP density. We first identified all 3kb windows of the genome for which there was no evidence of a recombination hotspot ($p>0.05$). We removed all putative coldspots containing more than 1% missing sequence.

To match each hotspot with a corresponding coldspot, we first estimated the GC and CpG content of each hotspot in a 3kb window centered on the hotspot peak center. For each hotspot, we then identified all coldspots on the same chromosome with a GC content within 0.5% of the hotspot GC content and CpG content within 0.1%. If more than one coldspot met these criteria, the coldspot matching the hotspot closest in terms of SNP density was chosen, as measured within a 20kb windows centered on the hotspots and coldspots. Using this method, we were able to identify a matched coldspot for 4,759 hotspots.

We extracted the DNA sequences associated with the 3kb windows around the center of the hotspots and coldspots. We tested all motifs with lengths between 3 and 10 base pairs inclusive. For each motif, we calculated the number of hotspots and coldspots having at least one copy of the motif, and calculated the significance of the difference using Fisher's Exact Test. Reported p-values were Bonferroni corrected within in each motif length class. We repeated the procedure twice: once having masked out repeat sequence (as defined by RepeatMasker), and once having masked out repeat sequence. The motifs reported in Table 1 show significance of $p < 0.01$ after Bonferroni correction in both repeat and non-repeat sequence.

Comparison of the distribution of recombination in dogs to human

Our recombination rate estimates suggest that dogs have a more uniform distribution through the genome than that observed in human (Figure S5A). However, we note that the estimated effective population size in dogs is higher than has been reported in human. In order to investigate if the observed distribution in dogs could be driven by this difference in effective population size, we conducted a simulation study. Using coalescent simulations, we simulated data under 3 different effective population sizes; 10,000, 20,000, and 30,000. The simulated dataset consisted of a 250kb region with a central 0.2cM hotspot, and a background rate of 0.0125 cM / Mb. Simulations for each effective population size were repeated 250 times. The results are shown in Figure S5B. As is clear, the effective population size can have a significant effect on the distribution of recombination, and hence it cannot be concluded that observed differences in the distribution of recombination between dog and human represent true differences in the underlying distribution of recombination.

H3K4me3 Chromatin Immunoprecipitation and sequencing (ChIP-seq)

Canine testes were obtained from routine neutering procedures from either the Cornell University Hospital for Animals, or the Tompkins County SPCA, and were stored for less than 12 hours in 1x PBS buffer in 4°C. The surrounding capsule and epididymis were removed and the testis cut up into 1 cm pieces with a scalpel blade, minced, collagenase treated, and added to Krebs buffer. The cell types were purified using gravity sedimentation, as a single cell suspension, according to the protocol described previously [39]. Briefly, the testis slurry was digested in collagenase and then trypsin to generate a single cell suspension, which was then run through a gravity sedimentation chamber at 4°C for several hours. The resulting sediment was fractionated and fractions examined for cellular content using a light microscope. The spermatocytes of specific prophase I stages were determined by their sizes and morphology. Cells were cross-linked in 1% formaldehyde and flash frozen.

Chromatin immunoprecipitation (ChIP) of histone H3 lysine 4 trimethylation (H3K4me3, using antibody ab8580 from Abcam) was performed using chromatin from approximately 2 million canine spermatogenic cells (pachytene and leptotene/zygotene spermatocytes were handled separately). ChIP was carried out according to the Myers laboratory protocol [40]. Prior to the incubation of chromatin with the antibody-coupled beads, one-tenth of the chromatin sample was removed to use as input for ChIP-seq. During the overnight incubation at 65°C to reverse cross-linking, 5 M NaCl and Proteinase K were added to the reaction. ChIP was validated through qPCR, with primers as described in Table S6.

Illumina ChIPseq libraries were prepared using Tru-Seq adaptors. Sequencing was performed on input and ChIP samples for both cell types using 150 bp paired-end sequencing from an Illumina HiSeq 2500 sequencer in Rapid Run Mode.

Reads were subsequently mapped to the canine reference genome using *bwa* [31] (0.6.2-r126). H3K4me3 peaks were called using *MACS* [34] assuming a reference genome size of 2.2e9 and a bandwidth of 150.

Analysis of biased gene conversion

Biased gene conversion is a proposed mechanism by which transmission of G/C alleles can occur preferentially over A/T alleles in the vicinity of double strand breaks associated with recombination (Figure S13A). To observe the effect of biased gene conversion, it was necessary to polarize SNP polymorphisms by the ancestral allele. This is complicated by the lack of reference genome for the fox. Therefore to assign ancestral alleles, we used an *ad hoc* approach.

To assign ancestral alleles in dog, we called genotypes in the fox at all sites called in the dog. We assigned the reference allele as ancestral if the fox was called as

homozygous for the reference allele, and assigned the alternative allele as ancestral if the fox was called as homozygous for the (dog) alternative allele. We required at least 5 reads and a genotype quality of at least 10 in the fox, and did not assign ancestral alleles at other sites.

For the converse process of assigning an ancestral allele for fox SNP polymorphisms, we extracted all positions in which the fox was heterozygotic with one of the alleles being the dog reference base. In this case, we took the ancestral allele as the dog reference base. We did not assign ancestral alleles when the fox was homozygotic for either the reference or alternative alleles.

Having defined ancestral alleles, we were able to investigate patterns of biased gene conversion. We define the 'skew' of AT->GC mutations relative to GC->AT mutations as the ratio of the number of observed polymorphisms observed in each class.

$$\text{Skew} = \frac{\#(AT \rightarrow GC\ polymorphisms)}{\#(GC \rightarrow AT\ polymorphisms)}$$

To investigate the properties of the 'skew' statistic, we performed a simulation study to investigate the effect of biased gene conversion. We used the SFS_CODE software package [41], which provides a highly flexible forward simulator that can model the effects of biased gene conversion. We simulated polymorphism data in 50 individuals within 5000 10kb regions, each containing a central hotspot with a 1kb with and a $4N_er$ across the hotspot of 17. We repeated the simulations both with and without biased gene conversion. In the biased gene conversion case, parameters were selected to match estimates regarding BGC obtained in the literature, such that 90% of recombination events result in a gene conversion with a mean tract length of 150bp. The allele bias was taken as 0.83, matching experimental sperm typing estimates [8]. We then estimated the #AT->GC / #GC->AT ratio averaged across the hotspots, using only sites segregating within the samples.

The results are shown in Figure S13B. In the absence of biased gene conversion, there is no change in the skew statistic in the vicinity of the hotspot. However, in the presence of biased gene conversion, a clear peak can be seen, qualitatively similar to the pattern observed around canine hotspots.

Our simulations therefore support the interpretation that pattern observed in Figure 3 could be the result of biased gene conversion. However, it is worth noting that the statistic we use can have a base-composition dependency. For example, a region containing few AT nucleotides will necessarily have fewer AT->GC mutations, which would depress the skew statistic. This is relevant for our simulations as over long periods of time the cumulative effect of biased gene conversion around evolutionarily stable hotspots would be to increase the local GC content. As such, this would ultimately influence our skew statistic via changes to the local base composition. Over moderate time spans, there is insufficient accumulation of GC to

alter the simulation result, and this in confirmed by simulations with longer burn-in periods (not shown). However, due to this base-composition dependency, the peak in the skew statistic observed in our simulations is not expected to represent the ultimate distribution under stationarity.

Genome annotations

Gene annotations and were downloaded from Ensemble in canFam2 coordinates. DNA repeat and CpG island were downloaded from the UCSC genome browser, also in canFam2 coordinates. These annotations were lifted to the coordinates of our reference build using the UCSC liftover tool (having generated the appropriate 'chain' files). Annotations that uniquely lifted over to our reference were kept (30,173 genes).

Sanger Sequencing of *PRDM9* in foxes

We sequenced the zinc-finger encoding exon 7 of *PRDM9* in an Island Fox, *Urocyon littoralis,* and a Culpeo (or Andean Fox), *Lycalopex culpaeus*. PCR sequencing was performed using the same procedure described in Axelsson *et al*. [13].

Supplementary References

36. The Broad Institute (2012) The canFam3.1 reference assembly. http://www.broadinstitute.org/ftp/pub/assemblies/mammals/dog/canFam3.1/
37. Wysoker A (2012) Picard. http://picard.sourceforge.net
38. Auton A, McVean G (2007) Recombination rate estimation in the presence of hotspots. Genome Res 17: 1219-1227.
39. Bellve AR (1993) Purification, culture, and fractionation of spermatogenic cells. Methods Enzymol 225: 84-113.
40. Pauli F, Myers R (2010) Myers Lab ChIP-seq Protocol, v041610.1 and v041610.2. http://myers.hudsonalpha.org/documents/Myers Lab ChIP-seq Protocol v041610.pdf
41. Hernandez RD (2008) A flexible forward simulator for populations subject to selection and demography. Bioinformatics 24: 2786-2787.
